# Hyperthermia Intensifies α-Mangostin and Synthetic Xanthones’ Antimalignancy Properties

**DOI:** 10.3390/ijms25168874

**Published:** 2024-08-15

**Authors:** Jakub Rech, Dorota Żelaszczyk, Henryk Marona, Agnieszka Gunia-Krzyżak, Paweł Żmudzki, Ilona Anna Bednarek

**Affiliations:** 1 Department of Biotechnology and Genetic Engineering, Faculty of Pharmaceutical Sciences in Sosnowiec, Medical University of Silesia, 40-055 Katowice, Poland; ibednarek@sum.edu.pl; 2 Department of Bioorganic Chemistry, Chair of Organic Chemistry, Faculty of Pharmacy, Jagiellonian University Medical College, 30-688 Krakow, Poland; dorota.zelaszczyk@uj.edu.pl (D.Ż.); henryk.marona@uj.edu.pl (H.M.); agnieszka.gunia@uj.edu.pl (A.G.-K.); 3 Department of Pharmaceutical Chemistry, Faculty of Pharmacy, Jagiellonian University Medical College, 30-688 Krakow, Poland; pawel.zmudzki@uj.edu.pl

**Keywords:** xanthones, synthetic xanthone derivatives, chemotherapy, hyperthermia, cytotoxicity, ovarian cancer, heat shock proteins, mangostin, metastasis, mitochondria

## Abstract

In order to improve naturally occurring xanthones’ anticancer properties, chemical synthesis is proposed. In this study, from eight novel xanthone derivatives coupled to morpholine or aminoalkyl morpholine, only the two most active ones were chosen. For additional enhancement of the anticancer activity of our tested compounds, we combined chemotherapy with hyperthermia in the range of 39–41 °C, from which the mild conditions of 39 °C were the most influencing. This approach had a profound impact on the anticancer properties of the tested compounds. TOV-21G and SC-OV-3 ovarian cell line motility and metastasis behavior were tested in native and hyperthermia conditions, indicating decreased wound healing properties and clonogenic activity. Similarly, the expression of genes involved in metastasis was hampered. The expression of heat shock proteins involved in cancer progression (Hsc70, HSP90A, and HSP90B) was significantly influenced by xanthone derivatives. Chemotherapy in mild hyperthermia conditions had also an impact on decreasing mitochondria potential, visualized with JC-1. Synthetic xanthone ring modifications may increase the anticancer activity of the obtained substances. Additional improvement of their activity can be achieved by applying mild hyperthermia conditions. Further development of a combined anticancer therapy approach may result in increasing currently known chemotherapeutics, resulting in a greater recovery rate and diminishment of the cytotoxicity of drugs.

## 1. Introduction

Currently, ovarian cancer is one of the most dangerous diseases affecting women, with reports of almost 324,398 new cases and 206,839 deaths in 2022 worldwide, and the estimation of 20,000 new diagnoses, and more than 12,000 deaths, in 2022 in the US alone [1,2]. Additionally, this type of cancer has a high recurrence rate and the possibility of developing drug resistance during initial chemotherapy, making it even harder to treat [3]. Recent treatment strategies involve chemotherapy combined with surgery, but due to the mentioned problems, combined strategies are commonly utilized, of which hyperthermia has the longest history of use. Cancer cells developed in the body are used to standard body conditions, and their manipulation has an impact on their behavior. In particular, hyperthermia possesses a strong effect on cancer cells, with a direct cytotoxic effect at high temperatures (42.5–43 °C) [4]. However, these temperatures are hard to obtain in vivo due to tissue and bloodflow differences. Mild hyperthermia (39 °C) is much easier to obtain but it still disrupts cell metabolism, and increases membrane permeability and drug intake, increasing treatment effectiveness. Additionally, in vivo it also affects the tumor microenvironment, decreasing oxygen and nutrient supply by bloodflow disruption, and simultaneously modulation of immune response to cancer cells. Hyperthermia is also postulated to hamper DNA repair processes. Despite this fact, the accurate mechanism of action of increased temperature on cancer cells is still uncovered [5]. In ovarian cancer therapy, hyperthermic intraperitoneal chemotherapy (HIPEC) was introduced and is currently ongoing many clinical trials, but the overall positive outcome is still a point of debate [6].

The most common ovarian cancer chemotherapeutics are synthetic platinum agents like carboplatin or cisplatin (**CIS**). These compounds crosslink DNA strands, creating complexes that disrupt replication and transcription, leading to cell death. Additionally on the membrane of **CIS**-treated cancer cells can be found a higher number of immunostimulatory molecules, making them easier for recognition of immune cells. Also, **CIS** disrupts DNA repair machinery [7,8]. These effects are very similar to the hyperthermia effect itself and can be even more intensified by the hyperthermia stimulus.

Currently, the interest in anticancer substances derived from natural fruits and plants is growing. One of the most promising are xanthone ring-based compounds which present a broad anticancer spectrum in vitro [9]. Most known are α-mangostin (**MAG**) and gambogic acid, which are broadly investigated for their properties. MAG inhibits cancer cell division, blocking their cell cycle, decreases their invasiveness and motility, and also reduces angiogenesis, reducing bloodflow. Additionally, MAG is recognized to induce reactive oxygen species (ROS) in cells, affecting their mitochondria and metabolism [10]. Their activity can be further improved by synthetic modifications swiftly creating many candidates for clinical use. Other published data also suggest the connection between the activity and structure of such compounds [11,12].

**Figure 1:**
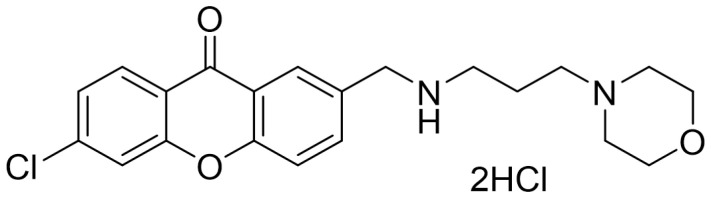
Structure of previously synthesized 6-chloro-2-(((3-morpholino propyl)amino)methyl)-9*H*-xanthen-9-one dihydrochloride [14].

In this work, we decided to focus on a xanthone scaffold modified with a morpholine ring. The first reason is that morpholine has been described as a privileged pharmacophore with a wide range of pharmacological activities due to different mechanisms of action, also with regard to anticancer activity [13]. In our previous work [14], we found that 6-chloro-2-(((3-morpholino propyl)amino)methyl)-9-xanthen-9*H*-one dihydrochloride (Figure 1) was one of the most promising anticancer compounds, showing antiproliferative and proapoptotic effects in five different cancer cell lines. The second reason for choosing the morpholine nucleus is that it can improve the aqueous solubility of lipophilic scaffolds [15].

**Figure 2:**
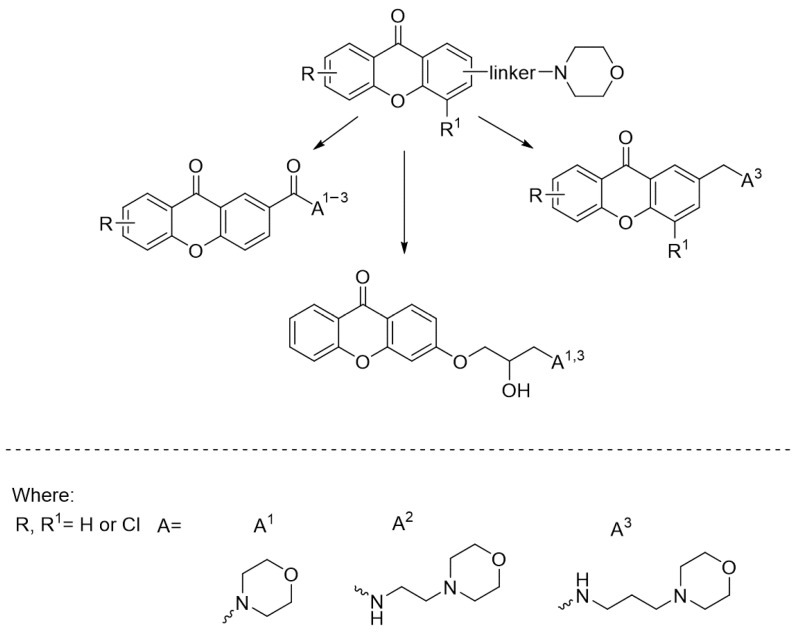
Proposed novel xanthone derivatives modifications.

Taking into account our previous studies and promising results, we aimed to further enhance the anticancer properties of novel xanthone derivatives by mild hyperthermia treatment. The aim of this study was to identify active anticancer compounds from a set of novel xanthone derivatives coupled to morpholine or aminoalkyl morpholine with different linkers (Figure 2) and assess their efficacy and the influence of mild hyperthermia conditions on ovary cancer cells in vitro.

We aimed to utilize the increased temperature as an additional enhancer of the anticancer activity of tested compounds. Initially, we screened eight novel xanthone derivatives for their cytotoxic properties and chose the most active ones. We compared the native and hyperthermia-treated cells’ migratory behavior to asses their malignancy. Additionally, mitochondria activity and total mass were evaluated by fluorescence staining. Also, the expression of heat shock proteins (HSPs) was assessed.

## 2. Results and Discussion

### 2.1. Chemistry

**Scheme 1:**
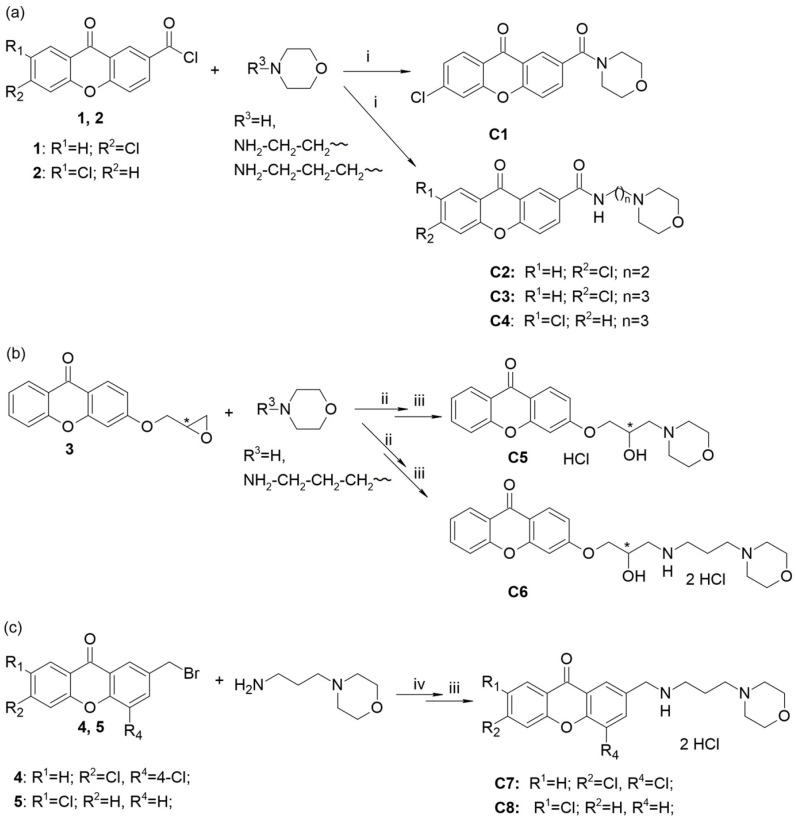
Synthesis of the new compounds (**a**) **C1**–**C4**, (**b**) **C5**–**C6**, (**c**) **C7**-**C8**. Reagents and conditions: (i) K_2_CO_3_, water, toluene, rt, 3 h; (ii) K_2_CO_3_, isopropanol, reflux, 3–6 h; (iii) ethanol saturated with HCl, rt, 30 min; and (iv) K_2_CO_3_, toluene, reflux, 4–8 h. The asterisk denote the chiral center.

The synthesis of final compounds **C1**–**C8** is illustrated in Scheme 1. The equimolar amount of an appropriate amine with xanthone starting materials reacted either in toluene/water (Scheme 1a) or toluene in the presence of K_2_CO_3_ (Scheme 1c) or in isopropanol (Scheme 1b) provided the xanthone derivatives as the powders. Amides (**C1**–**C4**) were tested as obtained, and amine derivatives (**C5**–**C8**) were converted into hydrochloric salts in order to improve their solubility. Compounds **C5** and **C6** were obtained as racemic mixtures. The spectral data of all compounds agreed well with the assigned structure.

### 2.2. Novel Xanthone Derivatives’ Cytotoxicity and Hyperthermia Conditions Establishment

Chemical synthesis is a widely known method of incorporating changes to well-described substances in order to obtain derivatives with stronger activity. This study estimated the influence of eight novel xanthone derivatives of morpholine or aminoalkyl morpholine on the growth inhibition (GI) of TOV-21G and SK-OV-3 ovarian cancer cell lines after 24 h (Table 1). For a better reflection of cancer behavior, we chose cell lines due to their different characteristics and **CIS** resistance, which is known to appear in patients with a history of platinum-containing chemotherapy. This initial screening allowed us to identify the most active compounds for further study.

Out of the eight tested compounds, two compounds were chosen (**C7** and **C8**) based on their overall lowest GI50. Both compounds were close analogs of a previously obtained compound (Figure 1, Table 2), differing in the position of the chlorine atom (**C8**) or in the presence of additional chlorine (**C7**) in the xanthone scaffold. **C7** exhibited activity at lower concentrations than **C8** against both cancer cell lines. Amide derivatives (**C1**–**C3**) were cytotoxic only against TOV-21G cells, while **C4** was active against both cell lines, but at high concentrations (over 100 μM). 3-(2-hydroxypropoxy)-xanthone coupled to morpholine (**C5**) was inactive against both cell lines in the performed assays, as was shown for its analog in our previous study [16], while its aminopropyl morpholine analog (**C6**) was cytotoxic at concentration over 80 μM, which excluded this compound from further study.

Additionally, the selected compounds were used in concentrations not toxic to the native L929 cell line (Supplementary materials, Table S1). This fact is important when designing new compounds and assessing their properties, as native healthy cell toxicity is not desired and may indicate unwanted side effects. Also, the route of administration and alternative drug delivery vehicles should be considered. The design of this study assumes hyperthermic intra-peritoneal infusion (HIPEC) as the administration route to prevent metastasis; thus, these concentrations are vital, but in order to further decrease the drug concentration and prolong drug release time we suggest incorporating these substances into drug delivery vehicles. Currently, vast interest is pointed towards biocompatible and biodegradable devices like polymers (PLGA, TMC, and PCL) and biopolymers (collagen and fibrin), providing an extended drug release time [17,18,19]. We suggest the use of exosomes, due to the additional possibility of targeted delivery, inclusion of additional adjuvants like miRNA, and reported enhanced silencing of HSP expression compared to free HSP inhibitors [20,21]. The use of exosomes as a drug delivery vehicle may also decrease the dose of xanthone derivatives which may be useful, especially for low-solubility derivatives.

**Figure 3:**
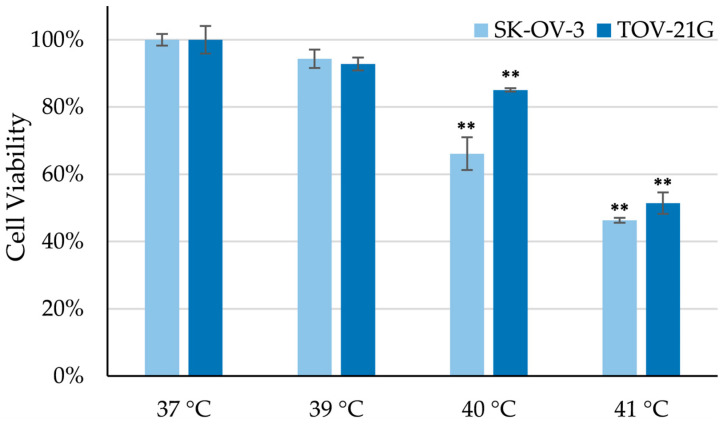
Effect of different hyperthermia temperatures on growth of SK-OV-3 and TOV-21G cell lines compared to growth in 37 °C set as 100%. ** *p* < 0.01 vs. untreated 37 °C negative control.

The second step was the estimation of hyperthermia’s influence on both cell lines and on compound activity. To assess the influence of changing environment temperature, we chose temperatures based on relevant data, ranging from 37 °C (native), 39 °C (mild), 40 °C, to 41 °C (strong hyperthermia) [22]. Due to the obtained results and our aim of the study to utilize hyperthermia as an additive and not the main action tool against the cancer cells, we chose 39 °C as most suited to this study (Figure 3). Additionally, it is reported that even performing hyperthermia procedures at higher temperatures does not guarantee that tumors will achieve an optimal thermal dose of 43 °C for 1h but the risk of the appearance of dangerous hot-spots increases [22]. The SK-OV-3 cell line was more susceptible to increasing temperature, especially over 40 °C.

After selecting hyperthermia and xanthone derivatives, we proceeded to test the chosen compounds, **CIS** and **MAG**, in native (37 °C) and hyperthermia conditions (39 °C) after 24 and 48 h of cell incubation. **CIS** was chosen due to being routinely used in chemiotherapy and **MAG** served as a natural xanthone reference compound. Calculated GI50, GI25, and GI10 are presented in Table 3. For further experiments, we chose 37 °C GI10 to treat cells.

### 2.3. Hyperthermia Increases the Long-Term Cytotoxicity of Novel Xanthone Derivatives

**Figure 4:**
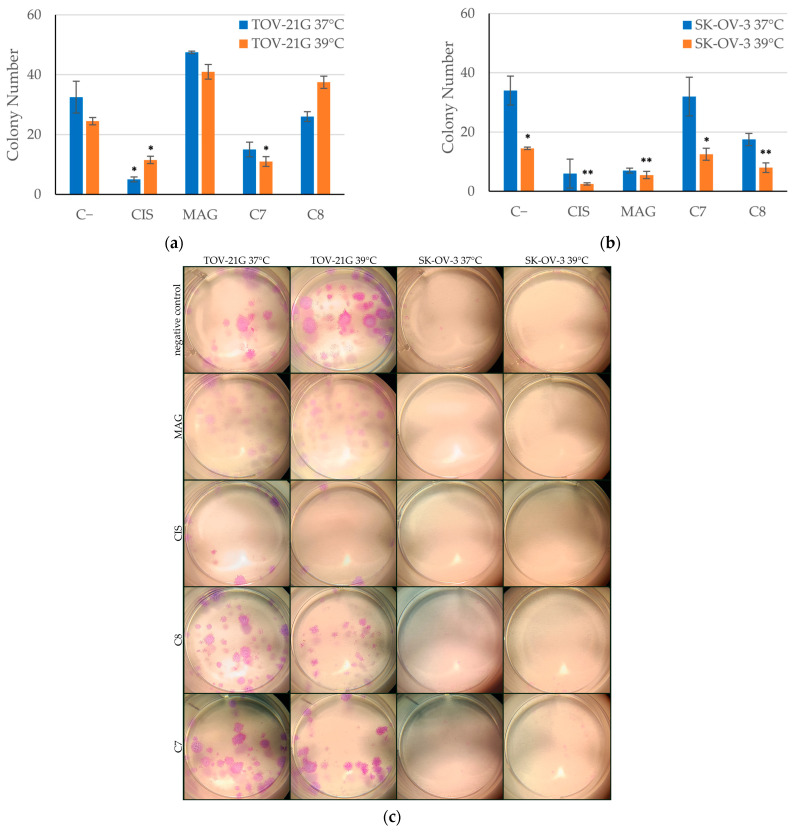
Hyperthermia and xanthone derivative treatment influence on the number of colonies formed of (**a**) TOV-21G and (**b**) SK-OV-3 cell line, (**c**) representative images of clonogenic assay. * *p* < 0.05; ** *p* < 0.01 vs. untreated 37 °C negative control.

The long-term cytotoxicity of the selected compounds and the hyperthermia influence were assessed with a colony formation assay. Detailed results are presented in Figure 4.

At 37 °C, compounds **C7**, **C8**, and **CIS** decreased the number of colonies of TOV-21G. A SK-OV-3 inhibitory effect was presented by compounds **C7**, **C8**, **MAG**, and **CIS**.

Similar to their hyperthermia response, the SK-OV-3 cells in every case of heat treatment presented decreased clonogenic potential. In the case of TOV-21G cells at 39 °C, only compound **C7** and **MAG** presented a moderate decrease in colony number. Heating TOV-21G cells treated with compound **C8** and **CIS** increased the mean colony number by 11.5 and 6.5, respectively, while hyperthermia itself decreased the colony number by 8, more efficiently than combined with **MAG** and **C7** treatment (6.5 and 4, respectively).

Metastasis involves a number of stages, one of which is the settling of cells in new niches and the continuation of growth [23]. Hyperthermia indicates a decreased colony-forming potential and a further decrease in the size of newly formed colonies, which leads to slower cancer recurrence. In the case of SK-OV-3 cells, the sensitivity to hyperthermia also provides a stronger response to contemporary chemotherapy stimuli. This is not an obligatory phenomenon and mostly depends on the type of cancer that is visible in the case of TOV-21G cells. However, hyperthermia alone seems to be safe and hampers the cells’ colony formation activity.

The MTT assay and colony formation assay are considered to measure the overall cell metabolism and division rate, but they depend on different mechanisms and measure different properties of cells in the end. The MTT assay is designated to measure mitochondrial enzymes, on which **CIS** has no direct impact, but it influences mitochondrial activity. Thus, **CIS** effectiveness in the MTT assay is lower, but it is superior to other substances in the colony forming assay. The **CIS** genotoxic and cytotoxic effect is based on the appearance of DNA adducts; thus, the initial **CIS** response may be lower, but the accumulation of such adducts over time may have an impact on cell viability by a gradual decrease in the concentration of proteins essential for cell survival. **CIS** also induces ROS production and, again, the changes in cell viability and ROS-induced damage accumulation will be more visible over time. These **CIS** properties can induce apoptosis and necrosis. **CIS** also disrupts the cell cycle, inducing cell cycle arrest in the G2/M phase [7].

### 2.4. Hyperthermia Induces Apoptosis

**Figure 5:**
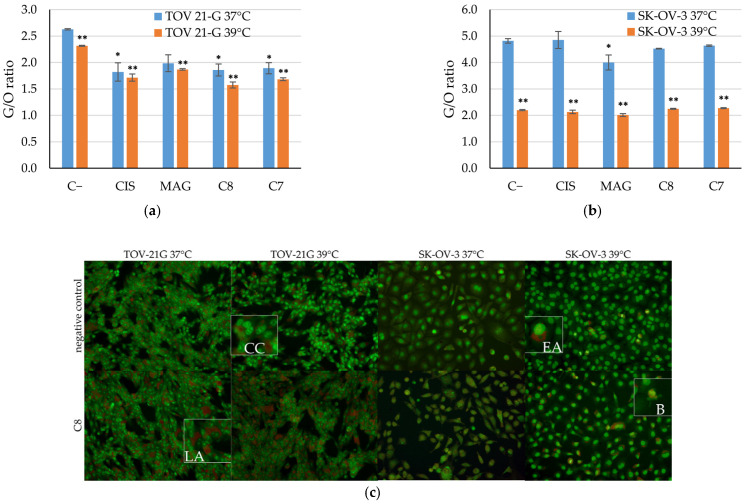
Hyperthermia and xanthone derivative treatment influences apoptosis. Calculated green/orange fluorescence ratio of (**a**) TOV-21G and (**b**) SK-OV-3 cell line. Representative images (**c**) of negative control and after 24 h of compound **C8** treatment of both cell lines. B—blebbing; CC—chromatin condensation; EA—early apoptosis; LA—late apoptosis. * *p* < 0.05; ** *p* < 0.01 vs. untreated 37 °C negative control.

To further investigate the cytotoxic influence of the tested compounds, the cells were stained with acridine orange/propidium iodide (AO/PI). This staining method allows for the assessment of cell viability and the detection of apoptosis based on the different properties of the dyes. AO penetrates intact cell membranes and stains all cells, whereas PI stains only cells with disrupted cell membranes. The total green fluorescence emitted by AO, divided by the orange fluorescence emitted by PI (G/O fluorescence ratio), indicates cell viability. In every tested condition, the green-to-orange fluorescence ratio was decreased in both cell lines compared to the untreated control. This indicates higher PI uptake, which points towards possible membrane damage and apoptosis [24]. While for TOV-21G these changes were more subtle, for SK-OV-3 cells the green/orange fluorescence ratio abruptly decreased in higher temperatures (Figure 5a,b). This effect is in line with the higher vulnerability of SK-OV-3 heated to 39 °C exposed by the MTT assay in Section 2.2. Additionally, the induction of apoptosis of the tested compounds is much higher than hyperthermia alone. Assumed additional stimulus provided by hyperthermia was obtained in the case of the TOV-21G cells. The SK-OV-3 cells’ apoptosis was induced strongly by hyperthermia alone with slight fluctuating changes caused by compound exposure. Also in the photographs, there are visible signs of blebbing, chromatin condensation, and increased orange fluorescence intensity (Figure 5c), indicating elevated apoptosis in cells [24]. Even though the HIPEC treatment is proposed to last around 90 min. and at higher temperatures in our study, even a shorter period of hyperthermia shock results in a decrease in cell viability and apoptosis induction [22]. Thus, prolonging the exposure time or repeating the treatment over a longer period would lead to higher anticancer properties and removal of present metastasis niches.

### 2.5. Hyperthermia Boosts Xanthones’ Anti-Migratory Effects

**Figure 6:**
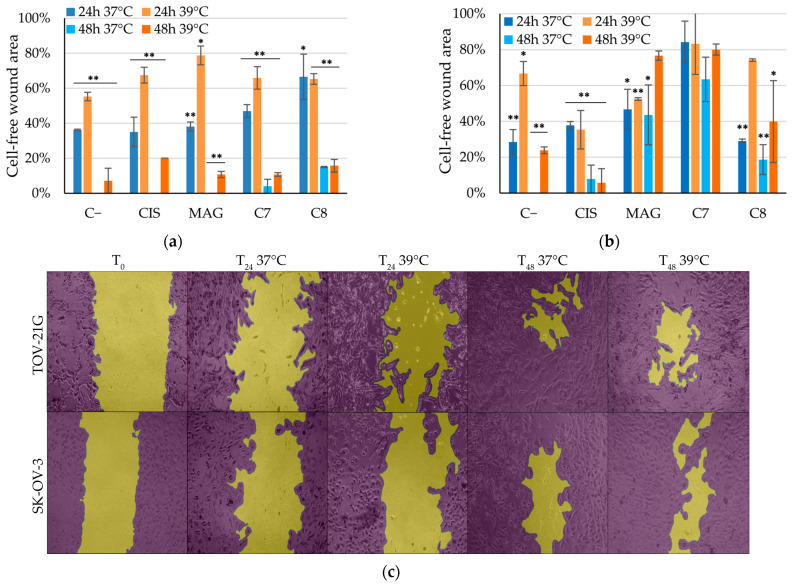
Wound area free from (**a**) TOV-21G and (**b**) SK-OV-3 cell growth after 24 and 48 h. Initial wound size was taken as a 100% wound size reference; (**c**) representative original images of the wound and overlayed wound size prediction by the program (purple/yellow) after **C7** treatment. * *p* < 0.05; ** *p* < 0.01 vs. initial wound size at T_0_.

A scratch assay (wound healing assay) was used to measure the migratory potential of cells, and the onward influence of xanthones and hyperthermia on this parameter. For precise measurement, a Python-based program was invented based on the idea of image entropy and possible division based on regions with different entropy values [25]. The precision of the program was adjusted for both cell lines due to their different morphologies. In Figure 6, the results are presented altogether with representative images of the invented program and its prediction. All evaluations were performed in comparison to the initial wound size at T_0_.

There is a clear connection between hyperthermia and a hampering influence on the migration of both cell lines. After 48 h, both TOV-21G and SK-OV-3 cells at 37 °C (negative control) filled the wound, whereas cells exposed to 39 °C for 1h still left around 10–20% of the wound open. Combining chemotherapy with hyperthermia induced a greater migration inhibition response in every case, except for **C8** for TOV-21G and **C7** for SK-OV-3. In these odd cases, the response was comparable to chemotherapy only. Despite this fact, the difference is visible in the migration of **C7** and 39 °C-exposed SK-OV-3 cells after 48 h, which is much larger than for chemotherapy alone after 48h. Similar results for **MAG** influence and xanthone derivative with heterocyclic ring modification were obtained in our previous study [9].

**CIS** has a very interesting behavior due to similar activity to the untreated TOV-21G control after 24 h and 48 h, but heat exposure increased its activity beyond this point and was the strongest migration inhibitor after 48 h. The rest of the tested substances had a higher initial activity which decreased during 48 h, but still, combined hyperthermia and chemotherapy, had higher potential than separate treatments.

In contrast, the SK-OV-3 cells being more susceptible to heat also reflects their migration ability, which is decreased even more by the addition of compounds **C7** and **C8**. After 48 h and 39 °C-exposure, the anti-migratory effect was decreased slower than for TOV-21G cells. Compound **C7** has a predominant anti-migratory effect on SK-OV-3 cells, which was prolonged and emphasized over 48 h by exposure to 39 °C.

### 2.6. Mitochondrial Mass and Membrane Potential

The next step of our research was to assess the influence of both hyperthermia and the xanthone derivatives on the mitochondrial mass and membrane potential with NAO (acridine orange 10-nonyl bromide) and JC-1 staining, respectively. NAO stains all cell mitochondria regardless of their state and membrane potential. Green fluorescence corresponding to total mitochondrial content in cells was measured. The JC-1 dye fluorescence wavelength is dependent on mitochondrial potential. In a standard state, the JC-1 probe is present in monomeric form and emits green fluorescence. When cells have high mitochondrial potential, the JC-1 forms J-aggregates, which results in an emission wavelength shift and the emission of red fluorescence. 

**Figure 7:**
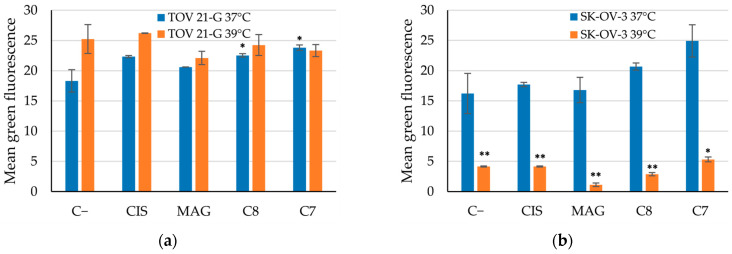
Average NAO staining green fluorescence corresponding to total mitochondrial mass of (**a**) TOV-21G and (**b**) SK-OV-3 cells. * *p* < 0.05; ** *p* < 0.01 vs. untreated 37 °C negative control.

We observed the contrary effect of hyperthermia, where it leads to an increment in the mitochondrial mass of TOV-21G cells but a decrement in the SK-OV-3 mitochondrial mass (Figure 7). A decrease in mass may also reflect the lesser thermal resistance of SK-OV-3 cells, as mentioned in Section 2.1.

Exposure of TOV-21G cells to chemotherapeutics in every case increased their total mitochondrial mass, with the greatest influence of compound **C7**. Similarly, SK-OV-3 treated cells also increased their mitochondrial content, especially when exposed to compound **C7**.

Combined hyperthermia and chemotherapy of TOV-21G cells in every case resulted in increased mitochondrial content compared to the 37 °C control. The highest mass was recorded when cells were exposed to **CIS** and the lowest when treated with **MAG**. SK-OV-3 cells with the 39 °C and **MAG** treatment also indicated the lowest mitochondrial mass. In this case, the lowest impact was reported by compound **C7**.

These results indicate that different cells respond in different ways to hyperthermia and hyperthermia itself can increase the response of cells to tested compounds (**CIS** and **MAG**) but also alter its final effect. These phenomena are visible in the case of compound **C8** on SK-OV-3 cells, where the compound treatment increases the overall mitochondrial mass, but compounds combined with hyperthermia additionally decrease the mass.

**Figure 8:**
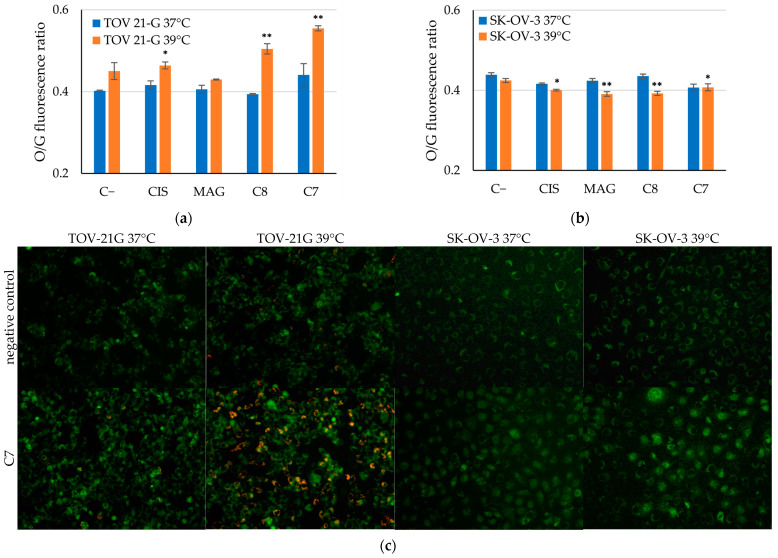
The mitochondrial membrane potential of (**a**) TOV-21G and (**b**) SK-OV-3, measured by JC-1 staining. Results are presented as orange-to-green fluorescence ratio (O/G ratio). Exemplary images (**c**) of JC-1 fluorescence after 24 h compound **C7** treatment. * *p* < 0.05; ** *p* < 0.01 vs. untreated 37 °C negative control.

The activity of mitochondria was assessed using JC-1 staining, which measures mitochondrial membrane potential. To account for compensatory mechanisms where decreased mitochondrial activity is offset by an increase in total mitochondrial mass, our findings were compared to previously documented total mitochondrial mass data [26]. In SK-OV-3 cells, the exposure led to a vast decrease in mitochondrial mass, but only a slight decrease in their membrane potential was observed (Figure 7a and Figure 8a). An opposite effect was observed for TOV-21G cells, (increased mass and increased activity) (Figure 7b and Figure 8b), particularly under heat treatment. This suggests that autophagy, a response to hyperthermia known to enhance thermoresistance, may be involved [27]. The lower thermotolerance of SK-OV-3 cells also supports this hypothesis (Figure 3).

Treatment of TOV-21G with compound **C7** has the greatest impact in increasing mitochondrial activity, while on the other hand, in the case of SK-OV-3, these substances decrease the mitochondrial activity the most (Figure 8c). In the case of TOV-21G, it seems that these substances increase both mitochondrial mass and membrane potential simultaneously, which may promote the survival of cells but also increase free radical synthesis.

Combined chemotherapy and thermotherapy of TOV-21G cells consistently resulted in an increased overall mitochondria potential compared to the negative conditions. **CIS**, **C8,** and **C7** had the strongest impact on membrane potential, particularly when combined with heat. Minimal changes were noted for **MAG**, even when compared with NAO staining. However, a decreased mitochondrial membrane potential was previously noted for **MAG** in other studies [28]. Additionally, hyperthermia improves the effect of the tested compounds on TOV-21G cells, contrary to SK-OV-3 cells, where impairment of mitochondrial activity was noted (Figure 8).

In summary, the tested compound activity depends on the cell line, either altering mitochondrial potential (TOV-21G) or affecting total mitochondrial mass (SK-OV-3). Previously, we also indicated increased levels of reactive oxygen species which can also contribute to changes in mitochondrial activity [9,26].

### 2.7. RNA Extraction and Real-Time RT PCR

**Figure 9:**
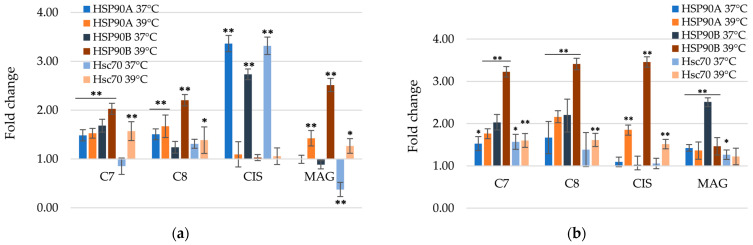
Fold change expression of HSP90A, HSP90B, and Hsc70 genes in (**a**) TOV-21G and (**b**) SK-OV-3, under native and hyperthermia conditions. **CIS** and **MAG** are used as reference compounds. * *p* < 0.05; ** *p* < 0.01 vs. untreated 37 °C negative control.

Three HSP genes were investigated for their involvement in different responses to the used therapeutics and hyperthermia (Figure 9a,b). HSPs are known chaperones of important protective behavior in response to cellular stress, e.g., temperature or xenobiotics [21,29]. Overexpression of HSPs may trigger multidrug resistance by the AKT/GSK3β/β-Catenin signaling pathway and their inhibition increases chemotherapy effectiveness [30]. This is also observed in our research, with the greatest changes in heat-induced HSP90B expression, but effective anticancer therapy should mainly aim to decrease the expression of HSPs [27]. HSP90A and HSP90B stabilize many proteins necessary for cancer survival such as HER2, AKT, and p53. HSP90A influences HIF-1α, increasing angiogenesis and cancer proliferation. Hsc70 also helps with proper protein folding to maintain homeostasis and is involved in the autophagy process. Thus, impaired action of HSPs would result in increased cell damage and, eventually, cancer cell death.

For TOV-21G in native conditions, **CIS**-induced expression of all HSPs was almost 3-fold, while both tested compound treatments led only to a slight increase. **MAG** had the opposite effect on HSPs, silencing their expression. Interestingly, hyperthermia decreased the **CIS**-induced influence on HSP expression, consequently silencing their expression. Similar activity was presented in SK-OV-3 cells treated with **MAG**. Reversely, SK-OV-3 treated with **CIS** at 39 °C increased levels of HSPs when compared to 37 °C. SK-OV-3 cells’ HSP expression was more constant and the effect of hyperthermia more predictable. In native conditions, all xanthone derivative treatments upregulated the expression of all HSPs, while **CIS** had no effect. Increased temperature additionally accelerated the expression of all HSPs, to the greatest extent for HSP90B, which is known to be temperature-induced. Also, in the case of **CIS,** hyperthermia strongly boosts the expression to similar levels to those of the tested derivatives. Only in the case of **MAG** was HSP90B hampered, and other gene expressions displayed no change.

## 3. Materials and Methods

### 3.1. Chemistry

#### 3.1.1. Chemicals and Equipment

The reagents used in the synthetic procedures are as follows: 2,4-dichlorobenzoic acid, 2,5-dichlorobenzoic acid, methyl 4-hydroxybenzoate, phenol, 2-chloro-4-methylphenol were provided by Fluorochem Ltd (Hadfield, UK), *p*-cresol, sodium sticks, (*R*/*S*)-epichlorohydrin were purchased from Alfa Aesar GmbH (Karlsruhe, Germany), whereas morpholine, 2-morpholinoethyl-1-amine, 3-morpholinopropyl-1-amine, thionyl chloride, N-bromo succinimide were supplied by Sigma-Aldrich (Saint-Louis, MO, USA). All reagents had at least 97% purity. Solvents (methanol, ethanol, toluene, hexane, isopropanol, *n*-propanol, acetone, and CCl_4_) and acids (conc. HCl and conc. H_2_SO_4_) as well as NaOH, NaHCO_3_, and K_2_CO_3_ were provided by Chempur (Piekary Śląskie, Poland). Deuterated solvents were purchased from Eurisotop (Saint-Aubin, France).

The melting points of the compounds were measured on the Electrothermal IA9300 apparatus (Electrothermal Engineering Ltd., Rochford, UK) and were given as uncorrected values. NMR spectra were recorded in DMSO-*d*_6_ or CDCl_3_ either on a Varian Mercury spectrometer (Varian, Inc., Palo Alto, CA, USA), operating at 300 MHz (^1^H NMR) or on a ROYALPROBE HFX using the JNM-ECZR500 at 500.16 MHz for ^1^H and 125.77 MHz for ^13^C NMR (JEOL USA Inc., Peabody, MA, USA) with a solvent as the internal standard. The multiplicity of ^1^H NMR signals was abbreviated as follows: s = singlet, d = doublet, dd = doublet of doublets, ddd = doublet of doublets of doublets, t = triplet, m = multiplet, and bs = broad singlet. Mass spectra were recorded on a UPLCMS/MS system consisting of a Waters ACQUITY UPLC (Waters Corporation, Milford, MA, USA) coupled to a Waters TQD mass spectrometer (electrospray ionization mode ESI-tandem quadrupole). Chromatographic separations were carried out using the Acquity UPLC BEH (bridged ethyl hybrid) 18 column, 2.1 mm × 100 mm with a 1.7 μm particle size, equipped with an Acquity UPLC BEH C18 Van Guard precolumn, which was 2.1 mm × 5 mm, with a 1.7 μm particle size. The column was maintained at 40 °C, and eluted under gradient conditions from 95% to 0% of eluent A over 10 min, at a flow rate of 0.3 mL min^−1^. Eluent A: water/formic acid (0.1%, *v*/*v*); eluent B: acetonitrile/formic acid (0.1%, *v*/*v*). Chromatograms were recorded using a Waters eλ PDA detector. Spectra were analyzed in the 200–500 nm range with a 1.2 nm resolution and a sampling rate of 20 points/s. MS detection settings of the Waters Xevo TQ-S Cronos mass spectrometer were as follows: source temperature 150 °C, desolvation temperature 350 °C, desolvation gas flow rate 600 L h^−1^, cone gas flow 100 L h^−1^, capillary potential 3.00 kV, and cone potential 30 V. Nitrogen was used for both the nebulizing and drying gas. The data were obtained in a scan mode ranging from 50 to 1000 *m*/*z* in 0.5 s time intervals. The compound chemical names were attributed by Chem BioDraw Ultra 21.0.0.28.

#### 3.1.2. The Synthesis and Characterization of Intermediate Products (**1**–**5**)

The acid chlorides 6-chloro-9-oxo-9*H*-xanthene-2-carbonyl chloride (**1**) and 7-chloro-9-oxo-9*H*-xanthene-2-carbonyl chloride (**2**) were obtained from the corresponding acids, which were prepared according to the previously reported procedure [31]. An appropriate acid (50 mmol) and thionyl chloride (100 mmol) were refluxed for 6 h in toluene (100 mL). Then, the solvent and unreacted thionyl chloride were distilled off, and the product was crystallized from the toluene/hexane.

##### 6-Chloro-9-oxo-9*H*-xanthene-2-carbonyl chloride (**1**)

White solid; m.p. 195–197 °C; C_14_H_6_Cl_2_O_3_; M.W. 293.10; ^1^H NMR (500 MHz,) δ ppm 8.67 (d, *J* = 2.2 Hz, 1 H, H-1), 8.31 (dd, *J* = 8.7, 2.2 Hz, 1 H, H-3), 8.17 (d, *J* = 8.6 Hz, 1 H, H-8), 7.89 (d, *J* = 2.0 Hz, 1 H, H-5), 7.72 (d, *J* = 8.7 Hz, 1 H, H-4), 7.54 (dd, *J* = 8.6, 2.0 Hz, 1 H, H-7) (Figure S1).

##### 7-Chloro-9-oxo-9*H*-xanthene-2-carbonyl chloride (**2**)

Light yellowish solid; m.p. 202–205 °C; C_14_H_6_Cl_2_O_3_; M.W. 293.10; ^1^H NMR (500 MHz, DMSO-*d*_6_) 8.61 (d, *J* = 2.3 Hz, 1 H, H-1), 8.28 (dd, *J* = 8.7, 2.3 Hz, 1 H, H-3), 8.03 (d, *J* = 2.7 Hz, 1 H, H-8), 7.88 (dd, *J* = 8.9, 2.7 Hz, 1 H, H-6), 7.70 (d, *J* = 8.9, 2 H, H-4, H-5) (Figure S2).

The synthesis and characterization of (*R/S*)-3-(oxiran-2-ylmethoxy)-9*H*-xanthen-9-one (**3**) were described elsewhere [32].

The methodology to obtain 2-(bromomethyl)-4,6-dichloro-9*H*-xanthen-9-one (**4**) was adopted from Eckstein et al. [33]. The synthetic procedure started with the conversion of 50 mmol of 2,4-dichlorobenzoic acid and 50 mmol of 2-chloro-4-methylphenol into their corresponding sodium salts. The next step was Ullman’s condensation, which was conducted in paraffin oil for 3h at 195–200 °C in the presence of a Cu/Cu_2_O catalyst. After cooling, toluene (50 mL) was added, and the mixture was filtered off. The raw product was dissolved in water (100 mL) and precipitated by adding diluted H_2_SO_4_. The precipitate was crystallized from ethanol. Afterward, the cyclization of the obtained product in concentrated H_2_SO_4_ (tenfold excess, *w*/*w*) was carried out in a boiling water bath for 2 h. Afterward, the mixture was poured into ice water (250 mL), and the precipitate was filtered off and washed with 10% NaHCO_3_. The crude 4,6-dichloro-2-methyl-9*H*-xanthone was crystallized from ethanol (m.p. 185–186 °C). An amount of 20 mmol of 4,6-dichloro-2-methyl-9*H*-xanthen-9-one was dissolved in CCl_4_ (50 mL); next, N-bromo succinimide (20 mmol) and catalytic amounts of benzoyl peroxide were added. The mixture was refluxed under visible light for 8 h. Then, the mixture was hot-filtered and evaporated to dryness to give a crude product purified by crystallization from toluene.

##### 2-(Bromomethyl)-4,6-dichloro-9*H*-xanthen-9-one (**4**)

Yellow solid, m.p. 204–206 °C; C_14_H_7_BrCl_2_O_2_; UPLC-MS purity 98.07%; M.W. 358.01; [M+H]^+^ calc.: 356.91, found: 356.88 *m*/*z*; ^1^H NMR (500 MHz, CDCl_3_) δ ppm 8.25 (d, *J* = 8.6 Hz, 1 H, H-8), 8.21 (d, *J* = 2.3 Hz, 1 H, H-1), 7.86 (d, *J* = 2.3 Hz, 1 H, H-3), 7.65 (d, *J* = 2.0 Hz, 1 H, H-5), 7.40 (dd, *J* = 8.6, 2.0 Hz, 1 H, H-7), 4.54 (s, 2 H, Ar-CH_2_) (Figures S3 and S4).

The synthesis and characterization of 2-(bromomethyl)-7-chloro-9*H*-xanthen-9-one (**5**) were reported earlier [33].

#### 3.1.3. The Synthesis of Final Compounds (**C1**–**C8**)

The synthesis procedures are illustrated in Scheme 1.

##### Amide Derivatives (**C1**–**C4**)

The method was adopted from a previous publication [34]. To a suspension of 5 mmol of morpholine or an appropriate aminoalkyl morpholine and 10 mmol of anhydrous K_2_CO_3_ in 5 mL of water and 5 mL of toluene, a solution of 7 mmol of the appropriate xanthone-2-carboxy chloride in 10 mL of toluene was added. The mixture was vigorously stirred for 3 h at room temperature. Then, the reaction mixture was heated to 80 °C, mixed for another 30 min., and left to cool down. The precipitate was filtered off and washed with a 10% solution of NaHCO_3_. Amides were recrystallized from toluene.

##### 3-(2-Hydroxypropoxy) Amine Xanthone Derivatives (**C5**–**C6**)

The applied synthetic procedure was previously described [35]. A suspension of 5 mmol of morpholine or aminopropyl morpholine and equimolar amount of (*R/S*)-3-(oxiran-2-ylmethoxy)-9*H*-xanthen-9-one in 25 mL of isopropanol was refluxed for 3–6 h. After aminolysis, the solvent was distilled off. The resulting amines were dissolved in diluted HCl, cleaned with charcoal, and then precipitated using NaOH solution. Amines were recrystallized from the mixture of ethanol/acetone and converted into hydrochloride salts using an excess of ethanol saturated with the gaseous HCl.

##### 2-Methyl Amine Xanthone Derivatives (**C7**–**C8**)

The methodology was adopted from Marona et al. [34]. A mixture of 7 mmol of aminopropyl morpholine and 5 mmol of an appropriate 2-bromomethyl-xanthone in 25 mL of toluene was refluxed for 5–6 h in the presence of 8 mmol of anhydrous K_2_CO_3_. The inorganic salt precipitate was filtered from the hot mixture and washed with hot toluene (2 × 5 mL). After cooling the filtrate, the target amine compound precipitated. It was filtered off and recrystallized from a mixture of toluene and heptane (5:1) (*v*/*v*). Bases were converted into salts (hydrochlorides) in n-propanol with an excess of EtOH saturated with HCl.

#### 3.1.4. Characterization Data for Final Compounds (**C1**–**C8**)

##### 6-Chloro-2-(morpholine-4-carbonyl)-9*H*-xanthen-9-one (**C1**)

White powder, m.p. 193.5–196 °C; C_18_H_14_ClNO_4_; UPLC-MS purity 98.02%; MW 343.76; [M+H]^+^ calc.: 344.07, found: 344.05 *m*/*z* (Figure S5); ^1^H NMR (300 MHz, DMSO-*d*_6_) δ ppm 8.19 (d, *J* = 8.8 Hz, 1 H, H-8), 8.16 (d, *J* = 2.3 Hz, 1 H, H-1), 7.87–7.95 (m, 2 H, H-3, H-5), 7.72 (d, *J* = 8.8 Hz, 1 H, H-4), 7.53–7.58 (m, 1 H, H-7), 3.45–3.75 (m, 8 H, 4×CH_2_ morph.) (Figure S6); ^13^C NMR (126 MHz, CDCl_3_) δ ppm 175.9, 168.8, 156.7, 156.3, 141.5, 134.6, 131.4, 128.3, 128.1, 125.8, 125.6, 121.3, 120.3, 119.1, 119.0, 119.0, 118.3, 118.2, 66.9 (2 C), 56.3 (2 C) (Figure S7).

##### 6-Chloro-*N*-(2-morpholinoethyl)-9-oxo-9*H*-xanthene-2-carboxamide (**C2**)

White powder, m.p. 188.5–190 °C; C_20_H_19_ClN_2_O_4_; UPLC-MS purity 100%; MW 386.83; [M+H]^+^ calc.: 387.11, found: 387.05 *m*/*z* (Figure S8); ^1^H NMR (300 MHz, DMSO-*d*_6_) δ ppm 8.76 (t, *J* = 5.6Hz, 1 H, NH), 8.69 (d, *J* = 2.3 Hz, 1 H, H-1), 8.30 (dd, *J* = 8.8, 2.3 Hz, 1 H, H-3), 8.21 (d, *J* = 8.7 Hz, 1 H, H-8), 7.90 (d, *J* = 2.0 Hz, 1 H, H-5), 7.74 (d, *J* = 8.8 Hz, 1 H, H-4), 7.55 (dd, *J* = 8.7, 2.0 Hz, 1 H, H-7), 3.56 (t, *J* = 9.4 Hz, 4 H, 2 × CH_2_-O), 3.37–3.45 (m, 2H, NH-CH_2_), 2.71 (t, *J* = 1.8 Hz, 2 H, CH_2_-N), 2.41 (t, *J* = 4.7 Hz, 4 H, 2 × N-CH_2_ morph) (Figure S9); ^13^C NMR (126 MHz, CDCl_3_) δ ppm 176.1, 165.8, 157.7, 156.2, 141.5, 134.8, 130.8, 128.2, 125.5, 124.6, 121.1, 120.3, 118.9, 118.3, 67.0 (2 C), 57.0, 53.5 (s, 2 C), 36.4 (Figure S10).

##### 6-Chloro-*N*-(3-morpholinopropyl)-9-oxo-9*H*-xanthene-2-carboxamide (**C3**)

White powder, m.p. 196–198 °C; UPLC-MS purity 98.97%; C_21_H_21_ClN_2_O_4_; MW 400.86; [M+H]^+^ calc.: 401.13, found: 401.08 *m*/*z* (Figure S11); ^1^H NMR (300 MHz, DMSO-*d*_6_) δ ppm 8.81 (t, *J* = 5.6 Hz, 1 H, NH), 8.68 (d, *J* = 2.3 Hz, 1 H, H-1), 8.30 (dd, *J* = 8.8, 2.3 Hz, 1 H, H-3), 8.21 (d, *J* = 8.8 Hz, 1 H, H-8), 7.89 (d, *J* = 1.8 Hz, 1 H, H-5), 7.73 (d, *J* = 8.8 Hz, 1 H, H-4), 7.55 (d, *J* = 8.8 Hz, 1H, H-7), 3.56 (t, *J* = 4.7 Hz, 4 H, CH_2_-O), 3.35–3.47 (m, 2 H, NH-CH_2_,) 2.23–2.44 (m, 6 H, CH_2_-N, 2 × N-CH_2_ morph), 1.70 (quin, *J* = 4.2 Hz, 2 H, CH_2_-CH_2_-CH_2_) (Figure S12); ^13^C NMR (126 MHz, CDCl*3*) δ ppm 175.9, 165.7, 157.7, 156.3, 141.4, 134.9, 131.1, 128.3, 125.4, 124.3, 121.1, 120.3, 118.9, 118.3, 66.9 (2 C), 59.2, 54.1 (2 C), 41.2, 23.9 (Figure S13).

##### 7-Chloro-*N*-(3-morpholinopropyl)-9-oxo-9*H*-xanthene-2-carboxamide (**C4**)

White powder, m.p. 172–173 °C; C_21_H_21_ClN_2_O_4_; UPLC-MS purity 100.00%; MW 400.86; [M+H]^+^ calc.: 401.13,found: 401.08 *m*/*z* (Figure S14); ^1^H NMR (500 MHz, CDCl_3_) δ ppm 8.59 (d, *J* = 2.3 Hz, 1 H-1), 8.47–8.52 (m, 1 H, NH), 8.35 (dd, *J* = 8.8, 2.3 Hz, 1 H, H-3), 8.28 (d, *J* = 2.6 Hz, 1 H, H-8), 7.67 (dd, *J* = 8.8, 2.6 Hz, 1 H, H-6), 7.55 (d, *J* = 8.8 Hz, 1 H, H-4), 7.46 (d, *J* = 8.8 Hz, 1 H, H-5), 3.78 (t, *J* = 4.6 Hz, 4 H, 2 × CH_2_-O), 3.57–3.64 (m, 2 H, NH-CH_2_), 2.57–2.61 (m, 2 H, CH_2_-N), 2.44–2.56 (m, 4 H, N- CH_2_ morph), 1.82 (quin, *J* = 5.9 Hz, 2 H, CH_2_-CH_2_-CH_2_) (Figure S15); ^13^C NMR (126 MHz, CDCl_3_) δ ppm 175.7, 165.6, 157.7, 154.5, 135.4, 135.0, 131.0 130.4, 126.3, 124.4, 122.6, 120.7, 119.9, 118.9, 66.9 (2 C), 59.2, 54.1 (s, 2 C), 41.2, 23.9 (Figure S16).

##### (R/S)-3-(2-Hydroxy-3-morpholinopropoxy)-9*H*-xanthen-9-one Hydrochloride (**C5**)

White powder, m.p. 210–212 °C; C_20_H_21_ClNO_5_; UPLC-MS purity 100%; salt MW 391.85; [M+H]^+^ calc.: 356.15, found: 356.21 *m*/*z* (Figure S17); ^1^H NMR (500 MHz, DMSO-d6) δ ppm 10.51 (br s., 1 H, NH^+^), 8.14 (dd, *J* = 7.9, 1.7 Hz, 1 H, H-8), 8.09 (d, *J* = 8.9 Hz, 1 H, H-1), 7.82 (ddd, *J* = 8.3, 7.1, 1.7 Hz, 1 H, H-6), 7.60 (d, *J* = 8.3 Hz, 1 H, H-5), 7.42–7.47 (m, 1 H, H-7), 7.19 (d, *J* = 2.4 Hz, 1 H, H-4), 7.05 (dd, *J* = 8.9, 2.4 Hz, 1 H, H-2), 6.06 (br s, 1 H, OH), 4.38–4.51 (m, 1 H, CH-OH), 4.08–4.21 (m, 2 H, O-CH_2_), 3.86–3.98 (m, 2 H, CH_2_-O morph.), 3.71–3.85 (m, 2 H, CH_2_-O morph.), 3.42–3.56 (m, 2 H, CH_2_-N), 3.04–3.28 (m, 4 H, 2 × N-CH_2_ morph) (Figure S18); ^13^C NMR (126 MHz, DMSO-*d*_6_) δ ppm 175.5, 164.5, 158.0, 156.2, 135.7, 128.2, 126.5, 125.0, 121.7, 121.7, 118.5, 115.7, 114.6, 101.8, 71.4, 63.8 (2 C), 59.1, 53.2 (2 C), 51.6 (Figure S19).

##### (R/S)-3-(2-Hydroxy-3-((3-morpholinopropyl)amino)propoxy)-9H-xanthen-9-one Dihydrochloride (**C6**)

White powder, m.p. 248–251 °C; C_23_H_30_Cl_2_N_2_O_5_; UPLC-MS purity 100%; salt MW 485.40; [M+H]^+^ calc.: 413.21, found: 413.24 *m*/*z* (Figure S20); ^1^H NMR (500 MHz, DMSO-*d*_6_) δ ppm 11.13 (br s, 1 H, NH^+^), 9.16 (br s, 1 H, NHH^+^), 8.89 (br s, 1 H, NHH^+^), 8.15 (dd, *J* = 7.8, 1.7 Hz, 1 H, H-8), 8.10 (d, *J* = 8.9 Hz, 1 H, H-1), 7.80–7.85 (m, 1 H, H-6), 7.61 (d, *J* = 8.4 Hz, 1 H, H-5), 7.45 (t, *J* = 7.7 Hz, 1 H, H-7), 7.18 (d, *J* = 2.4 Hz, 1 H, H-4), 7.06 (dd, *J* = 8.9, 2.4 Hz, 1 H, H-2), 5.99 (br s, 1 H, OH), 4.22–4.32 (m, 1 H, CH-OH), 4.13–4.21 (m, 2 H, Ar-O-CH_2_), 3.89–3.98 (m, 2 H, CH_2_-O morph.), 3.76–3.83 (m, 2 H, CH_2_-O morph.), 3.34–3.40 (m, 2 H, CH-CH_2_-NH), 2.95–3.24 (m, 8 H, NH-CH_2_, CH_2_-N, 2 × CH_2_-N morph.), 2.07–2.16 (m, 2 H, CH_2_-CH_2_-CH_2_) (Figure S21); ^13^C NMR (126 MHz, DMSO-*d*_6_) δ ppm 175.5, 164.5, 158.0, 156.2, 135.7, 128.2, 126.5, 125.0, 121.7, 118.5, 115.7, 114.6, 1018, 71.1, 65.2 (2 C), 63.6, 53.5, 51.5 (2C), 49.8, 45.0, 20.1 (Figure S22).

##### 4,6-Dichloro-2-(((3-morpholinopropyl)amino)methyl)-9*H*-xanthen-9-one Dihydrochloride (**C7**)

Yellowish powder, m.p. 282–283 °C; C_21_H_24_Cl_4_N_2_O_3_; UPLC-MS purity 99.47%; salt MW 494.23; [M+H]^+^ calc.: 421.11, found: 421.15 *m*/*z* (Figure S23); ^1^H NMR (500 MHz, DMSO-*d*_6_) δ ppm 11.29 (br s, 1 H, NH^+^), 9.57 (br s, 2 H, NH_2_^+^), 8.32–8.33 (m, 1 H, H-1), 8.30 (d, *J* = 2.2 Hz, 1 H, H-3), 8.17 (d, *J* = 8.5 Hz, 1 H, H-8), 7.94 (d, *J* = 1.9 Hz, 1 H, H-5), 7.57 (dd, *J* = 8.5, 1.9 Hz, 1 H, H-7), 4.29 (br s, 2 H, Ar-CH_2_), 3.75–3.96 (m, 4 H, 2 × CH_2_-O), 3.34–3.40 (m, 2 H, NH-CH_2_), 3.11–3.21 (m, 2 H, N-CH_2_), 2.93–3.07 (m, 4 H, 2 × N-CH_2_ morph.), 2.08–2.19 (m, 2 H, CH_2_-CH_2_-CH_2_) (Figure S24); ^13^C NMR (126 MHz, CDCl_3_) δ ppm 175.2, 156.0, 150.7, 141.3, 137.6, 135.2, 128.2, 125.4, 124.0, 122.9, 122.7, 120.0, 118.4, 67.1 (2 C), 57.5, 53.9 (2 C), 52.9, 48.1, 26.7 (Figure S25).

##### 7-Chloro-2-(((3-morpholinopropyl)amino)methyl)-9*H*-xanthen-9-one Dihydrochloride (**C8**)

White powder, m.p. 284–286 °C; C_21_H_25_Cl_3_N_2_O_3_; UPLC-MS purity 98.62%; salt MW 459.79; [M+H]^+^ calc.: 387.15, found: 387.17 *m*/*z* (Figure S26); ^1^H NMR (500 MHz, DMSO-*d*_6_) δ ppm 11.42 (br s, 1 H, NH^+^), 9.62 (br s, 2 H, NH_2_^+^), 8.35 (d, *J* = 2.2 Hz, 1 H, H-8), 8.11 (dd, *J* = 8.7, 2.3 Hz, 1 H, H-3), 8.09 (d, *J* = 2.3 Hz, 1 H, H-1), 7.90 (dd, *J* = 9.0, 2.2 Hz, 1 H, H-6), 7.72–7.75 (m, 2 H, H-4, H-5), 4.23–4.32 (m, 2 H, Ar-CH_2_), 3.73–3.98 (m, 4 H, 2 × CH_2_-O), 3.34–3.41 (m, 2 H, NH-CH_2_), 2.94–3.23 (m, 6 H, CH_2_-N, N-CH_2_ morph.), 2.16 (quin, *J* = 7.5 Hz, 2 H, CH_2_-CH_2_-CH_2_) (Figure S27); ^13^C NMR (126 MHz, DMSO-*d*_6_) δ ppm 175.5, 156.2, 154.7, 138.3, 136.1, 129.5, 129.1, 128.7, 125.4, 122.7, 121.4, 121.1, 119.3, 63.6 (2 C), 53.4, 51.3 (2C), 49.5, 44.3, 20.18 (Figure S28).

### 3.2. Chemicals for Biological Evaluations

Agarose, cisplatin, crystal violet, DAPI (4′,6-diamidino-2-phenylindole dihydrochloride), DMSO (dimethyl sulfoxide), α-mangostin, para-formaldehyde, and triton X-100 were purchased from Sigma-Aldrich (Saint Louis, MO, USA). All media and chemicals used for in vitro cell cultivation (DMEM, FBS, phosphate-buffered saline (PBS), trypsin–EDTA, Gel-Red, and PenStrep) were purchased from Gibco, ThermoFisher Scientific (Dublin, Ireleand). Xanthone stock solutions were prepared by dissolving xanthones in DMSO at a concentration of 10 mM each and frozen at −20 °C. α-mangostin (24 mM) and cisplatin (10 mM) stock solutions were prepared in methanol and 0.9% NaCl, respectively. Directly before assays, stock solutions were thawed and diluted to the desired concentration in DMEM.

### 3.3. Cell Culture and Treatment Conditions

In this study, TOV-21G (obtained from ATCC^®^ CRL-11730™) and SK-OV-3 (obtained from ATCC^®^ HTB-77™) ovarian cancer cell lines were used. Cells were grown in DMEM High Glucose with L-glutamine (Gibco, ThermoFisher Scientific, Dublin, Ireleand), supplemented with 10% FBS (PAN Biotech, Aidenbachch, Germany) and 1% PenStrep (Gibco, ThermoFisher Scientific, Dublin, Ireleand). Cells were grown in a HeraCell Heraeus (Hanau, Germany) cell incubator at 37 °C and 5% CO_2_. These cells were chosen due to their different origins, properties, and responses to therapy (TOV-21G—ovarian clear cell adenocarcinoma, primary origin of tumor cells; SKOV-3—ovarian serous cystadenocarcinoma, metastatic origin of tumor cells). For all assays, the cells were grown overnight, and fresh media containing xanthones at GI10 were added the following day.

Hyperthermia treatment was conducted by placing cells in a Panasonic MCO-19AIC (Kadoma, Japan) incubator set at 39 °C and 5% CO_2_ for 1h immediately after adding a pre-warmed medium containing xanthones.

### 3.4. Cell Viability Assay

Cell viability was assessed with the MTT microplate assay. Cells were seeded at an initial density of 15,000 per well on a 96-well microplate (SARSTEDT, Nümbrecht, Germany) and left overnight. Next, the media were removed and new media containing xanthones in serial dilution in the range of 200–1.076 μM were added. After 24 h/48 h of treatment, media were removed and cells were rinsed with PBS (Gibco, ThermoFisher Scientific, Dublin, Ireleand). After that, 100 μL of 0.5mg/mL MTT (Sigma, ThermoFisher Scientific, Dublin, Ireleand), in DMEM without phenol red (Gibco, ThermoFisher Scientific, Dublin, Ireleand), was added to each well and incubated at 37 °C for 2h. After incubation, the media were removed and 150 μL of DMSO/isopropanol (1:1) mixture was added to each well. Absorbance was measured with a Labtech LT-5000 Plate Reader (Uckfield, UK) microplate reader at 570 nm with a 630 nm reference filter. The concentrations inhibiting growth by 50% (GI50), 25% (GI25), and 10% (GI10) were calculated using equation (T − Tc)/(C − Cc) × 100, where T is the absorbance of the tested concentration of the selected compound, measured at 570 nm; Tc is the background absorbance of the tested concentration of the selected compound, measured at 630 nm; C is the negative (untreated) control absorbance, measured at 570 nm; and Cc is the background absorbance of the negative (untreated) control, measured at 630 nm. The GI50, GI25, and GI10 were calculated as the xanthone concentration that caused, respectively, 50%, 25%, and 10% growth inhibition.

### 3.5. Clonogenic Assay

The long-term influence of xanthone treatment on cancer cells was assessed with the clonogenic assay. Cells were seeded in 12-well plates (SARSTEDT, Nümbrecht, Germany) at 600 cells per well, and after 24 h media were replaced with media containing xanthone at GI10. The media with drugs in were changed every day. After 7 days (TOV-21G) and 14 days (SK-OV-3), media containing xanthones were removed, cells were rinsed with PBS, fixed in 4% paraformaldehyde containing 0.5% crystal violet, and then photographed. DMEM was used as a control group.

### 3.6. Scratch Assay

Xanthones’ influence on cell motility was estimated with a scratch assay (wound migration (healing) assay). The cells were seeded at 100,000 (TOV-21G) or 150,000 (SK-OV-3) per well on a 24-well microplate (SARSTEDT, Nümbrecht, Germany). After 24 h, when cells reached around 90% confluency, using a sterile 100 μL pipette tip a wound was made by dragging the tip over the cell’s monolayer. Then, unattached cells were removed by rinsing wells with PBS and then DMEM with xanthones at GI10 was added to each well. Cells were allowed to grow, with photographs taken at 24 h and 48 h after the treatment. The wound area was estimated using a self-developed Python program with human supervision (source code available in [36]). To evaluate cells, the disc size and the threshold values were as follows: for TOV-21G, 20 and 92 and for SK-OV-3, 50 and 92, respectively.

### 3.7. AO/PI, NAO, and JC-1 Staining

Fluorescence staining of cells was conducted on 24-well microplates (SARSTEDT, Nümbrecht, Germany). A total of 50,000 cells of each line were seeded on each well and left overnight. After this period, the cells were treated with tested substances and hyperthermia, as described before. The next day, the media were aspirated and cells were washed three times in PBS. Then, an appropriate amount of staining solution was added to each well and incubated at 37 °C. The staining solutions were prepared from stocks (AO and PI 1mg/mL in PBS; NAO 50 μM in DMSO; and JC-1 1 mg/mL in DMSO). Before staining cells, the dyes were diluted to working concentration (AO and PI 10 μg/mL in PBS; NAO 5 μM in culture media w/o supplements; and JC-1 10 μg/mL in culture media w/o supplements) at 37 °C with vortexing and forward filtration with 0.45 µm filter discs to remove any residue. Cells were then stained in darkness for 15 min. All images were taken by a Nikon Eclipse Ti (Nikon Instruments Inc., Melville, NY, USA) in 30 min and analyzed by NIS-Elements AR software (Nikon Instruments Inc., Melville, NY, USA; 64bit v.3.22.15 (build 738), LO). The green (AO)-to-orange (PI) fluorescence ratio was used to calculate cell viability. Mean NAO green fluorescence was used as equivalent to total mitochondrial mass. The JC-1 orange-to-green fluorescence ratio was used to present the mitochondrial membrane potential.

### 3.8. RNA Extraction and Real-Time™ RT PCR

For extraction and purification of total RNA, the GeneMATRIX Universal DNA/RNA/Protein Purification Kit (EUR_X_, Gdansk, Poland) was used according to the manufacturer protocol. The concentration and purity of the obtained extracts were assessed with a spectrophotometer (260 nm, BioPhotometer, Eppendorf) and band visualization on 2% agarose gels stained with GelRed.

To determine gene expression, a Real-Time™ RT-PCR method with SYBR Green dye was used. All experiments were run on a Mx3000P thermal cycler (Stratagene, San Diego, CA, USA). The first strand was obtained with an NG dART RT kit (EUR_X_, Gdansk, Poland), followed by Real-Time™ PCR using an SG/ROX qPCR Master Mix (EUR_X_, Gdansk, Poland) according to the manufacturer protocol. Temperatures were as follows: first strand synthesis 50 °C for 60 min, termination 85 °C for 5 min; Real-Time™ PCR initial denaturation 95 °C for 10 min, 40 cycles of 94 °C for 15 s, and 55 °C for 30 s, and 72 °C for 30 s, followed by 72 °C for 10 min and a dissociation curve protocol in range of 95–60 °C, lasting around 30 min. Starter sequences were as follows: HSP90A forward: 5′GTCCTGTGCGGTCACTTAGC3′; reverse: 5′ACTGGGCAATTTCTGCCTGA3′; HSP90B forward: 5′GTACGGATGGTCTGGCAACA3′; reverse, 5′GTCTCTGATCAGCGGGTGTC3′; Hsc70 forward: 5′TTATTGGAGCCAGGCCTACAC3′; reverse: 5′GCGACATAGCTTGGAGTGGT3′; HPRT1 forward: 5′CCTGGCGTCGTGATTAGTGA3′; reverse: 5′CGAGCAAGACGTTCAGTCCT3′.

Gene expression fold change was calculated using the ∆∆Cq method. Untreated cell cultures served as the control group. HPRT1 (gene coding for hypoxanthine phosphoribosyltransferase 1) was treated as the reference gene.

### 3.9. Statistics

Quantitative data were compared by Student’s *t*-test. For multiple comparisons, ANOVA with a post hoc Tukey HSD (Honestly Significant Difference) test was used; *p* < 0.05 (*) and *p* < 0.01 (**) were considered significant. All calculations were performed with Statistica v. 12 software.

## 4. Conclusions

In our study, the mild hyperthermia conditions indicated a major influence on ovarian cancer cells. The two used cell lines, TOV-21G and SC-OV-3, of miscellaneous origin and characteristics responded differently to the hyperthermia stimulus. Additionally, our idea of combined anticancer therapy, similar to the idea of clinically used HIPEC, promotes cancer cell death and inhibits cancer cells’ mobility and metastasis potential. These two novel xanthone derivatives (**C7** and **C8**) need further research to broadly unveil their promising anticancer properties. These results indicate that the anticancer mechanism of action of the tested substances is based on changes in mitochondria, namely their overall mass and membrane potential. Previously tested xanthone derivatives displayed similar proapoptotic and antiproliferative properties by inducing ROS generation in mitochondria [9]. Changes in the expression of metalloproteinases (MMP9 and MMP2), tissue inhibitor of metalloproteinases 1 (TIMP1), cathepsin D (CTSD), VEGF, HIF1α, ICAM-1, vascular cell adhesion protein 1 (VCAM-1), homing cell adhesion molecule (HCAM, CD44), and E- cadherin (CDH1) were also noted. The disruption of these genes involved in invasion and metastasis may be also correlated with changes in HSP proteins, decreasing cell survival, which should be further investigated. These changes may be also driven by extracellular communication by exosomes, known for carrying cargo influencing cell behavior and malignancy. Exosomes were also found to be carriers of various mRNAs involved in cancer progression; thus, it would be also vital to verify their influence on cell lines.

These novel xanthones present promising anticancer properties; nevertheless, the detailed mechanism of action is still unclear and should be further investigated.

## Figures and Tables

**Table 1:** GI50 of TOV-21G cells treated with novel xanthone derivatives.

Compound No.	TOV-21G GI50 [μM]	SK-OV-3 GI50 [μM]
**C1**	15.58	>150
**C2**	126.43	>150
**C3**	134.20	>150
**C4**	101.63	141.80
**C5**	>150	>150
**C6**	82.87	87.93
**C7**	16.17	28.30
**C8**	48.34	67.37

**Table 2:** The chemical structure of representative xanthone derivatives used in the study.

**Compound**	**Synthetic xanthone derivative structures**
**C7**	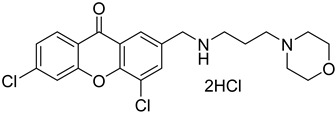
**C8**	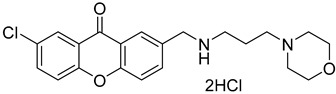
**Reference compound structures**	
**MAG**	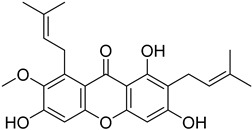
**CIS**	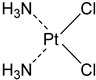

**Table 3:** GI50, GI25, and GI10 after 24 and 48 h in 37 °C and 39 °C.

		TOV-21G						SK-OV-2					
		GI50 [μM]		GI25 [μM]		GI10 [μM]		GI50 [μM]		GI25 [μM]		GI10 [μM]	
		24 h	48 h	24 h	48 h	24 h	48 h	24 h	48 h	24 h	48 h	24 h	48 h
37 °C	**C7**	16.17	15.77	10.94	11.01	7.81	8.16	28.30	16.35	18.03	10.63	11.87	7.19
	**C8**	48.34	32.20	23.01	19.75	7.81	12.28	67.37	53.13	45.12	33.21	31.78	21.26
	**MAG**	10.24	10.85	5.24	6.16	2.25	3.66	67.31	38.84	50.25	32.62	40.02	28.89
	**CIS**	46.56	54.22	31.51	28.46	22.49	13.01	27.30	9.91	12.00	5.33	2.82	2.58
39 °C	**C7**	25.14	16.16	13.39	9.65	6.34	5.74	27.08	26.19	15.43	15.15	8.41	8.53
	**C8**	49.04	22.77	24.22	15.41	9.32	10.98	59.18	52.59	27.45	25.13	8.45	8.65
	**MAG**	33.72	24.27	21.56	18.94	14.27	15.75	38.53	37.22	28.38	29.97	22.29	25.62
	**CIS**	65.11	20.21	26.28	11.91	2.98	6.93	99.86	37.13	38.10	18.46	1.05	7.25

## Data Availability

The raw data supporting the conclusions of this article will be made available by the authors on reasonable request.

## Supplementary Materials

The following supporting information can be downloaded at: https://www.mdpi.com/article/10.3390/ijms25168874/s1.
