# Supplementary material for: Hyperthermia Intensifies α-Mangostin and Synthetic Xanthones’ Antimalignancy Properties
Source: Int J Mol Sci. 2024 Aug 15;25(16):8874. doi: 10.3390/ijms25168874 (PMC11354364; doi:10.3390/ijms25168874)
Supplement: Supplementary file 1 [file ijms-25-08874-s001.zip › ijms-3115851-supplementary.pdf]

Hyperthermia intensifies gambogic acid and synthetic xanthones' antimalignancy properties.

Jakub Rech [1,*], Dorota Żelaszczyk [2], Henryk Marona [2], Agnieszka Gunia-Krzyżak[2], Paweł Żmudzki[3] and Ilona Bednarek [1]

[1] Department of Biotechnology and Genetic Engineering, Faculty of Pharmaceutical Sciences in Sosnowiec, Medical University of Silesia, 40-055 Katowice, Poland; jrech@sum.edu.pl (J.R), ibednarek@sum.edu.pl (I.B.)
[2] Department of Bioorganic Chemistry, Chair of Organic Chemistry, Faculty of Pharmacy, Jagiellonian University Medical College, 30-688 Krakow, Poland; dorota.zelaszczyk@uj.edu.pl (D.Z.); henryk.marona@uj.edu.pl (H.M.); agnieszka.gunia@uj.edu.pl (A.G-K)
[3] Department of Pharmaceutical Chemistry, Faculty of Pharmacy, Jagiellonian University Medical College, Krakow, 30-688 Krakow, Poland; pawel.zmudzki@uj.edu.pl (P.Z.)
[*] Correspondence: jrech@sum.edu.pl

Intermediate products

Compound 1

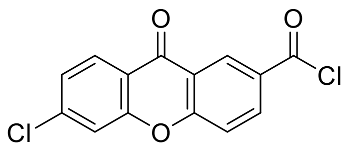

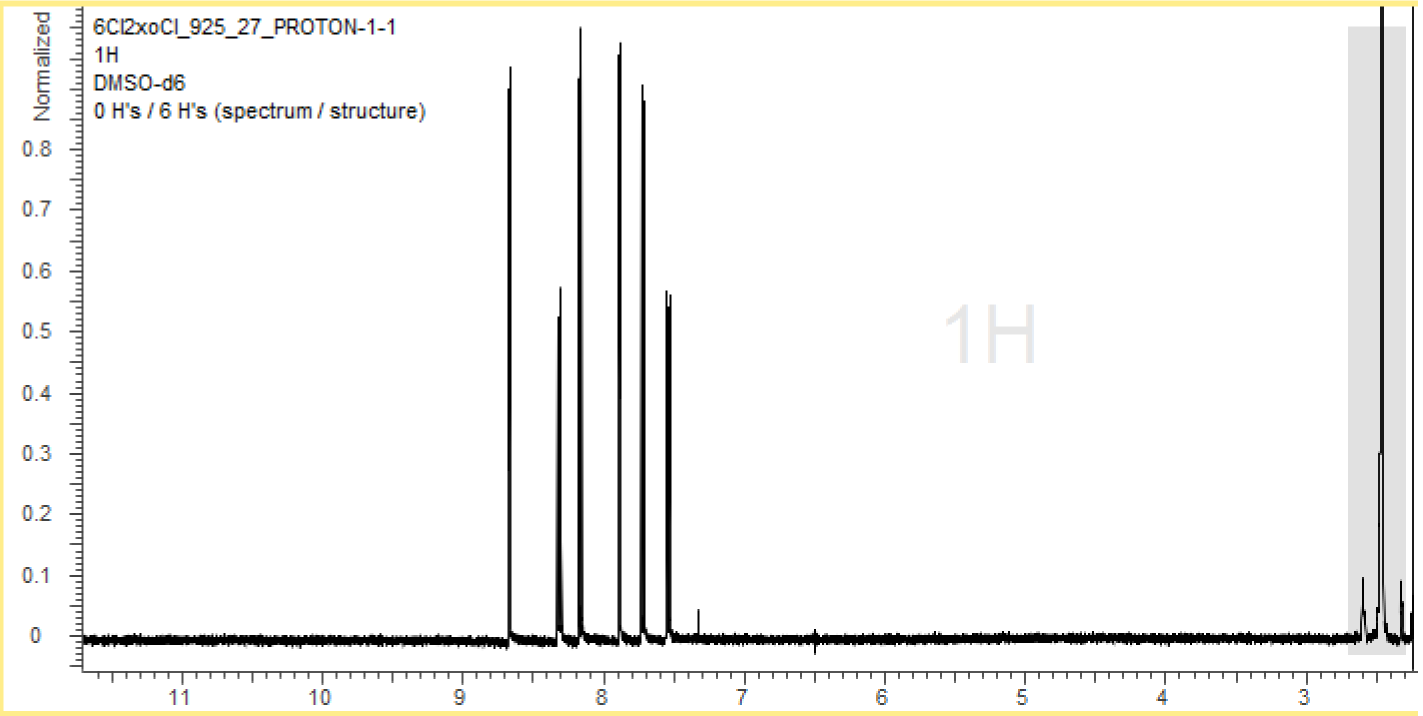

Figure S1

Compound 2

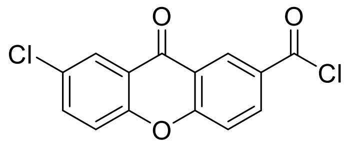

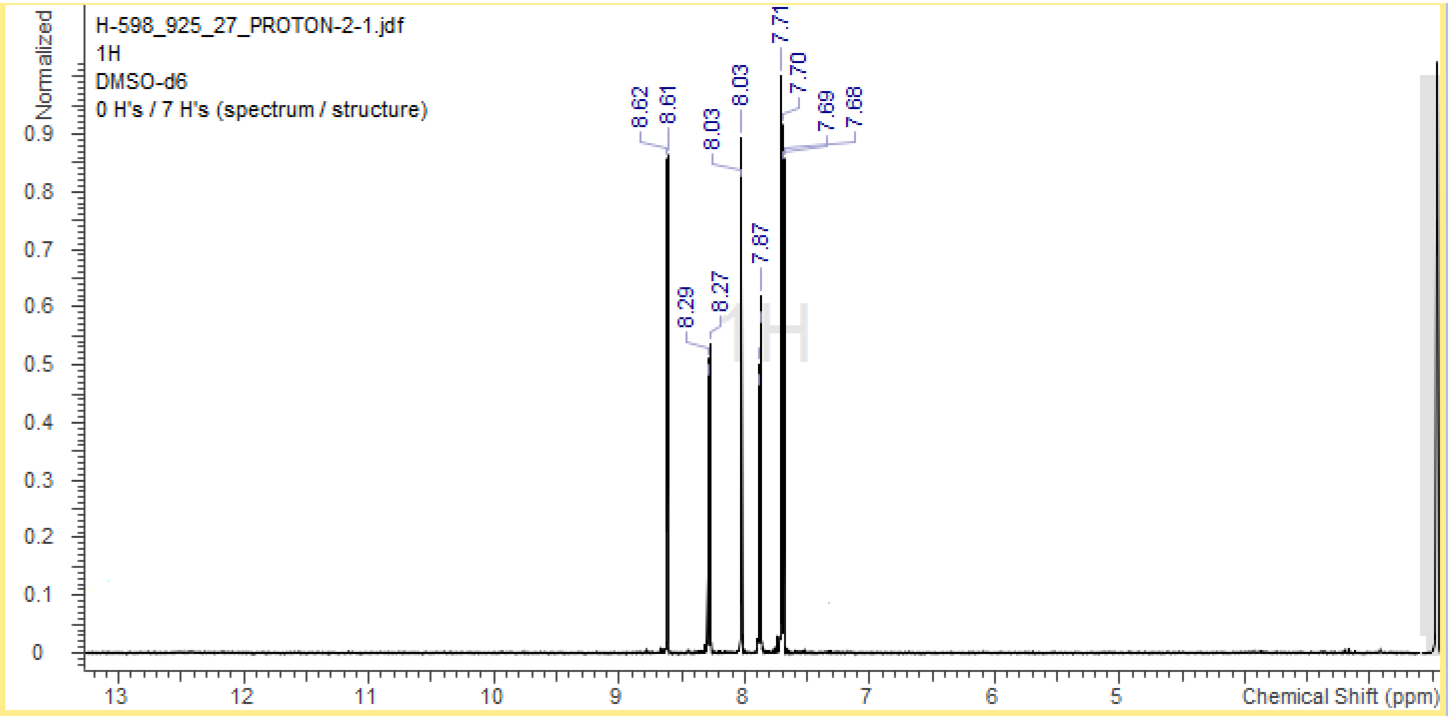

Figure S2

Compound 4

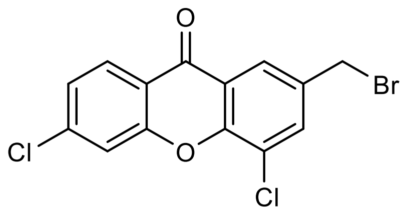

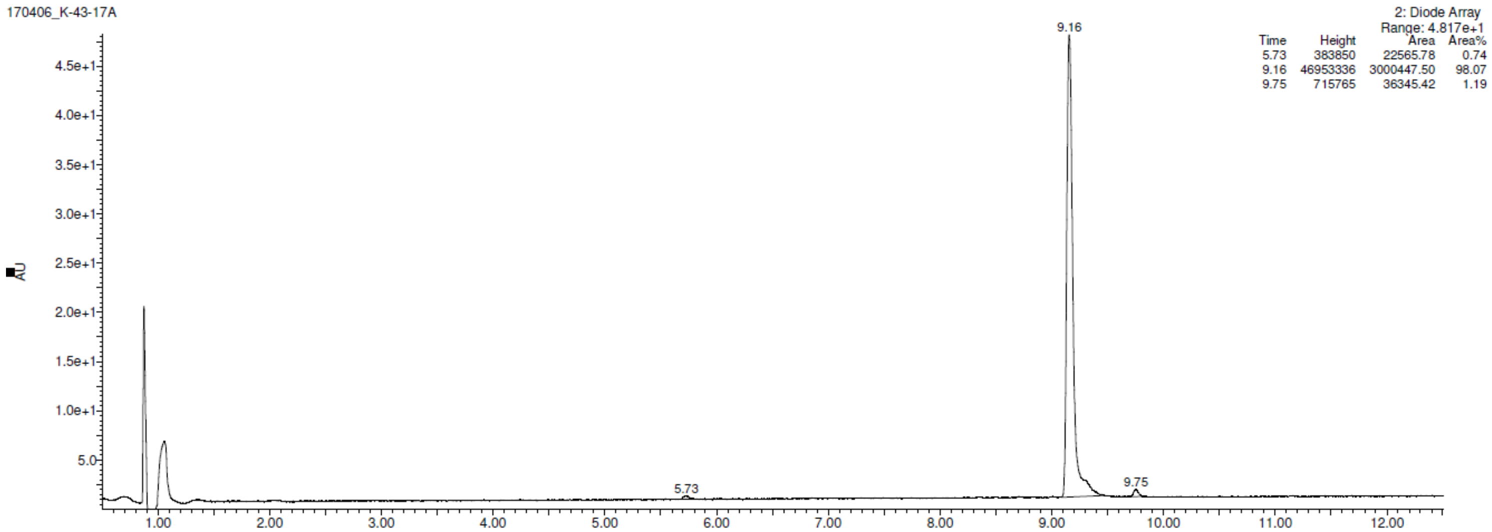

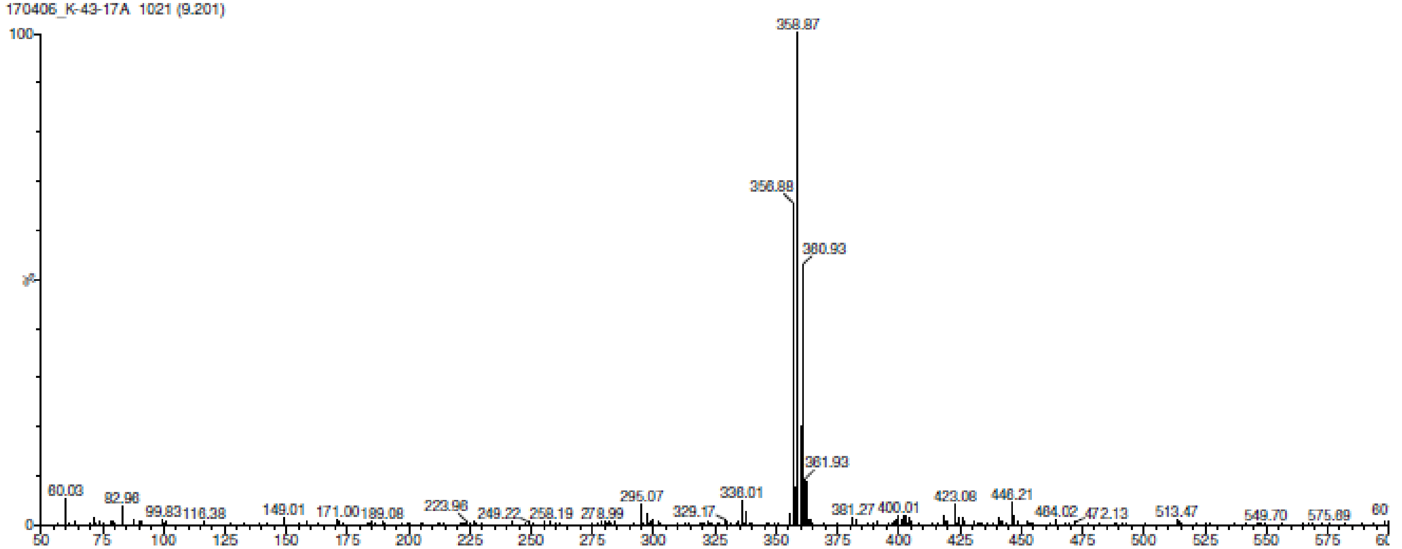

Figure S3

Compound 4

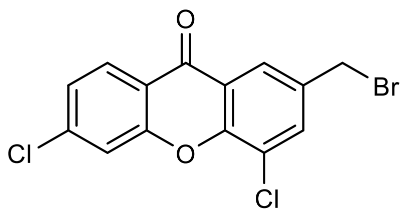

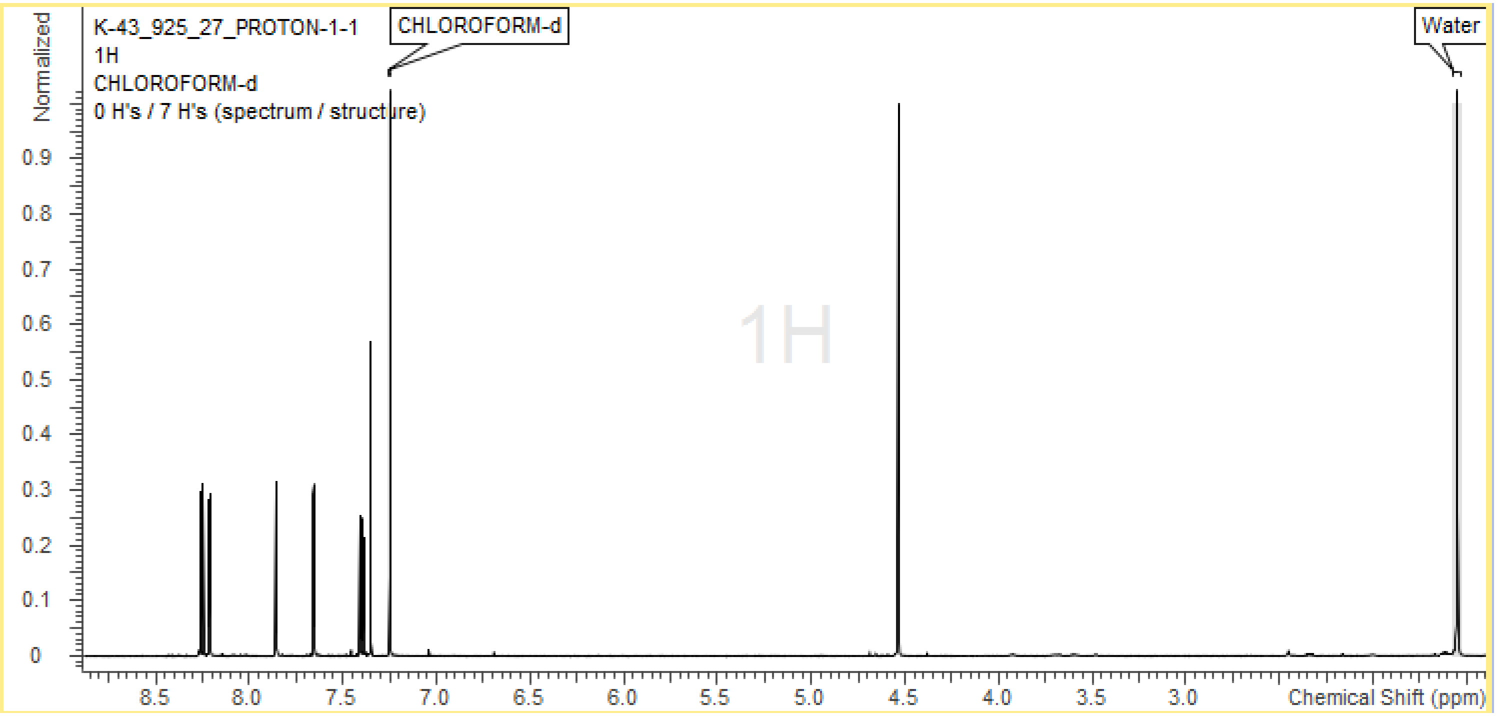

Figure S4

Final compounds

C1

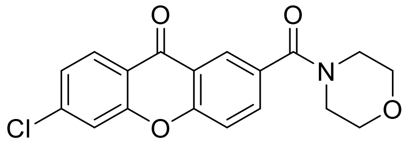

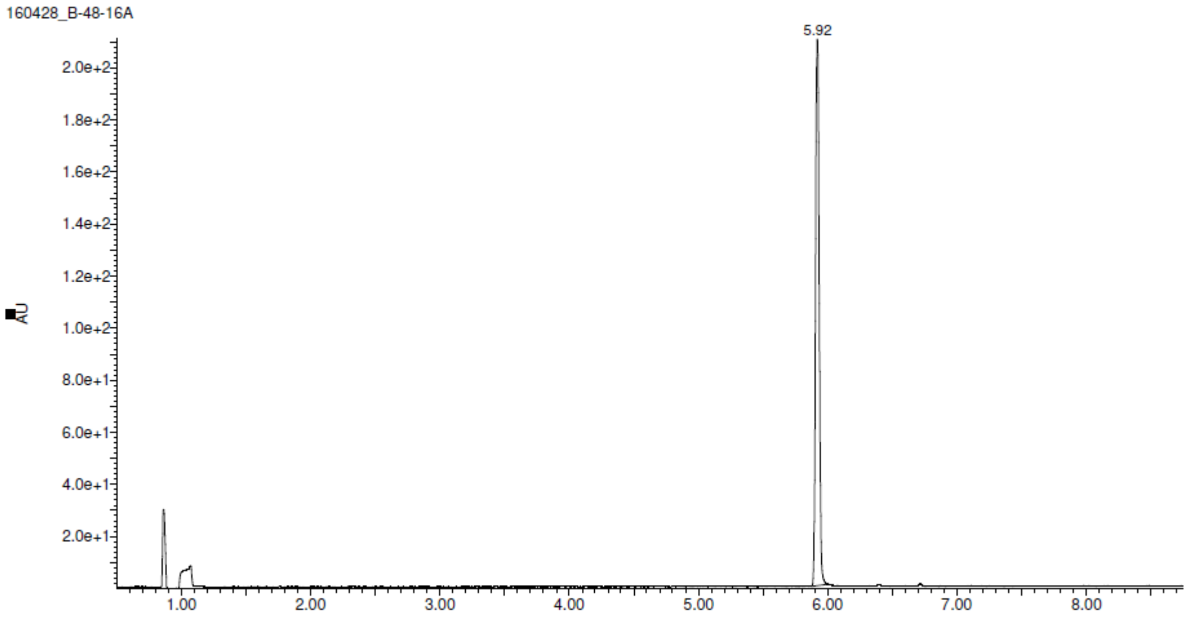

2: Diode Array
Range: 2.11e+2

Time	Height	Area	Area%
5.92	209975728	6850069.50	98.02
8.99	4384871	138584.27	1.98

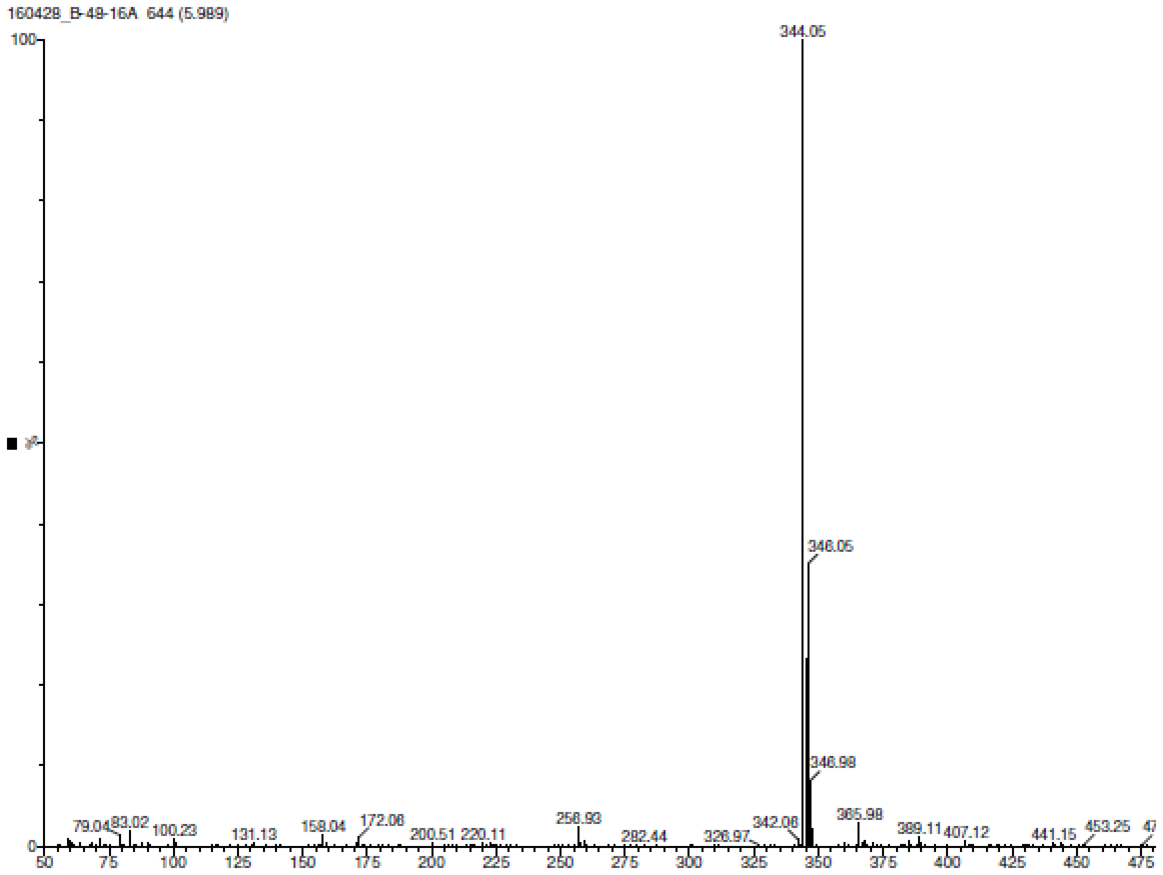

Figure S5

C1

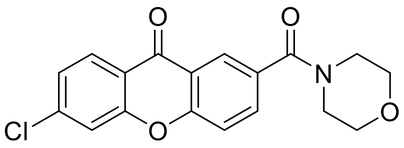
O
O
N
O
Cl
O

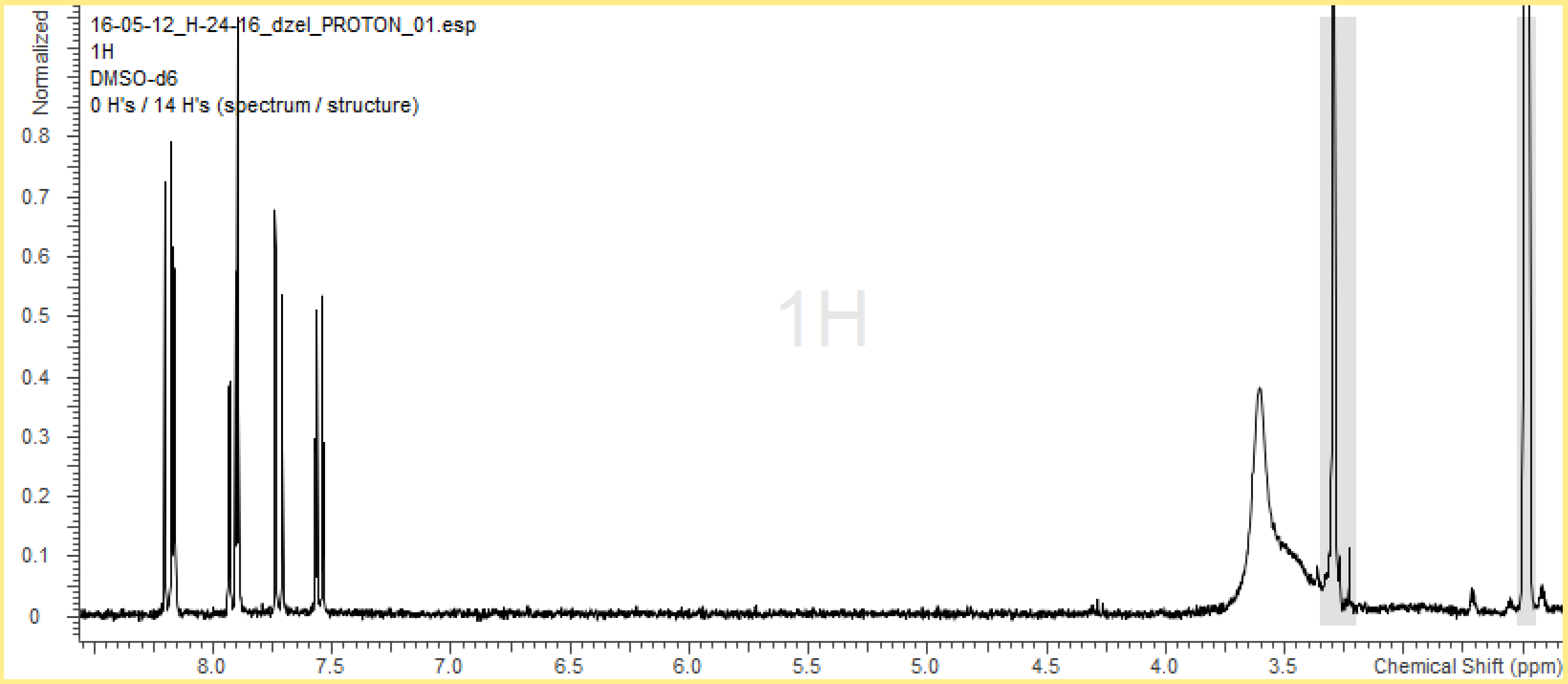
16-05-12_H-24-16_dzel_PROTON_01.esp
1H
DMSO-d6
0 H's / 14 H's (spectrum / structure)
Normalized
0.8
0.7
0.6
0.5
0.4
0.3
0.2
0.1
0
1H
8.0
7.5
7.0
6.5
6.0
5.5
5.0
4.5
4.0
3.5
Chemical Shift (ppm)

Figure S6

C1

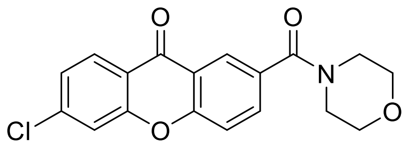

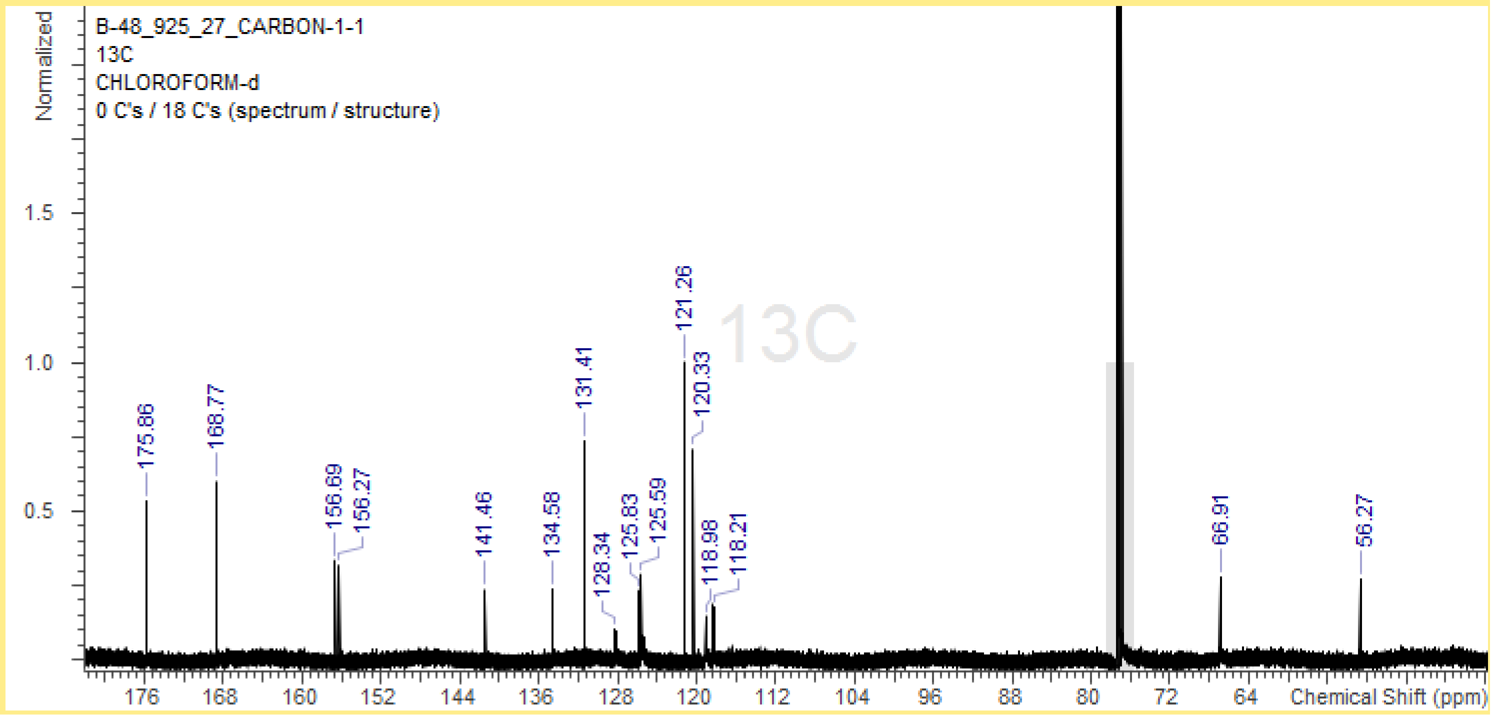
B-48_925_27_CARBON-1-1
13C
CHLOROFORM-d
0 C's / 18 C's (spectrum / structure)
Normalized
175.86
168.77
156.69
156.27
141.46
134.58
131.41
128.34
125.83
125.59
121.26
120.33
118.98
118.21
66.91
56.27
13C
Chemical Shift (ppm)

Figure S7

C2

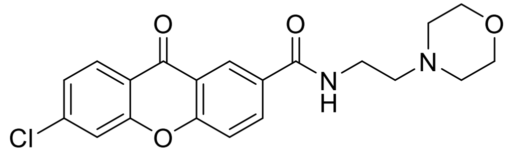
O
O
N
H
N
O
Cl
O

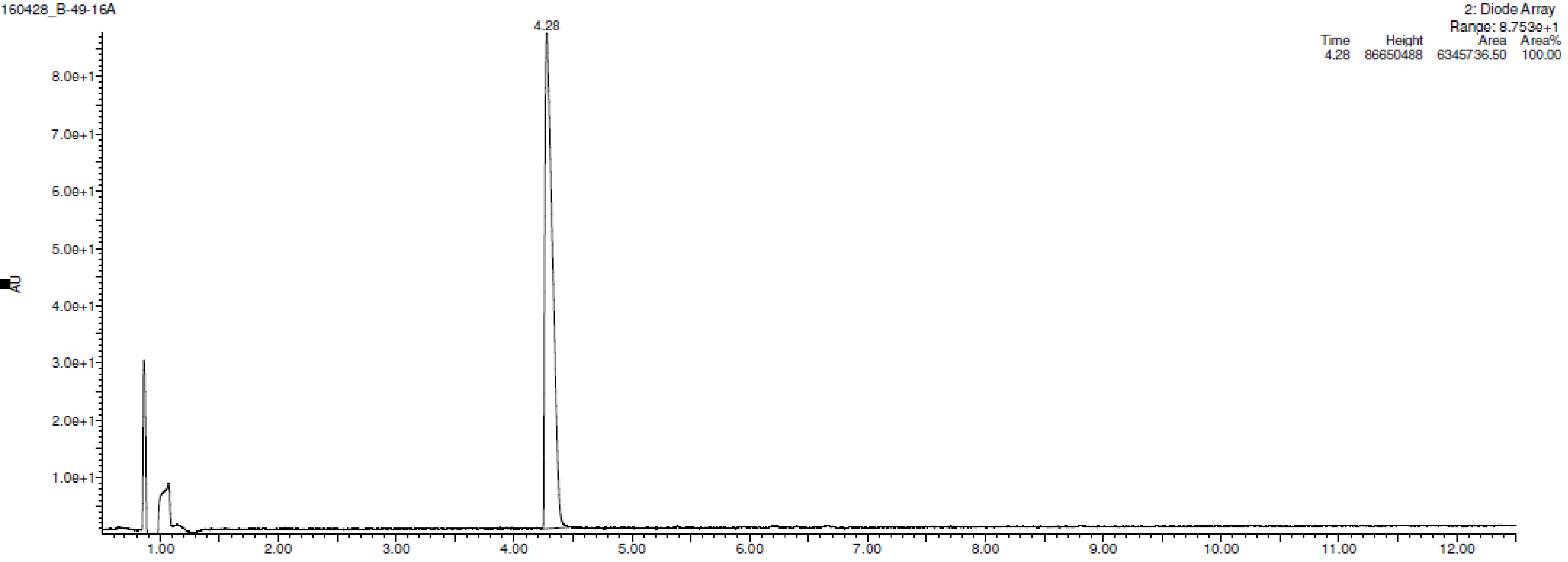
160428_B-49-16A
4.28
2: Diode Array
Range: 8.753e+1
Time Height Area Area%
4.28 86650488 6345736.50 100.00
AU
8.0e+1
7.0e+1
6.0e+1
5.0e+1
4.0e+1
3.0e+1
2.0e+1
1.0e+1
1.00
2.00
3.00
4.00
5.00
6.00
7.00
8.00
9.00
10.00
11.00
12.00

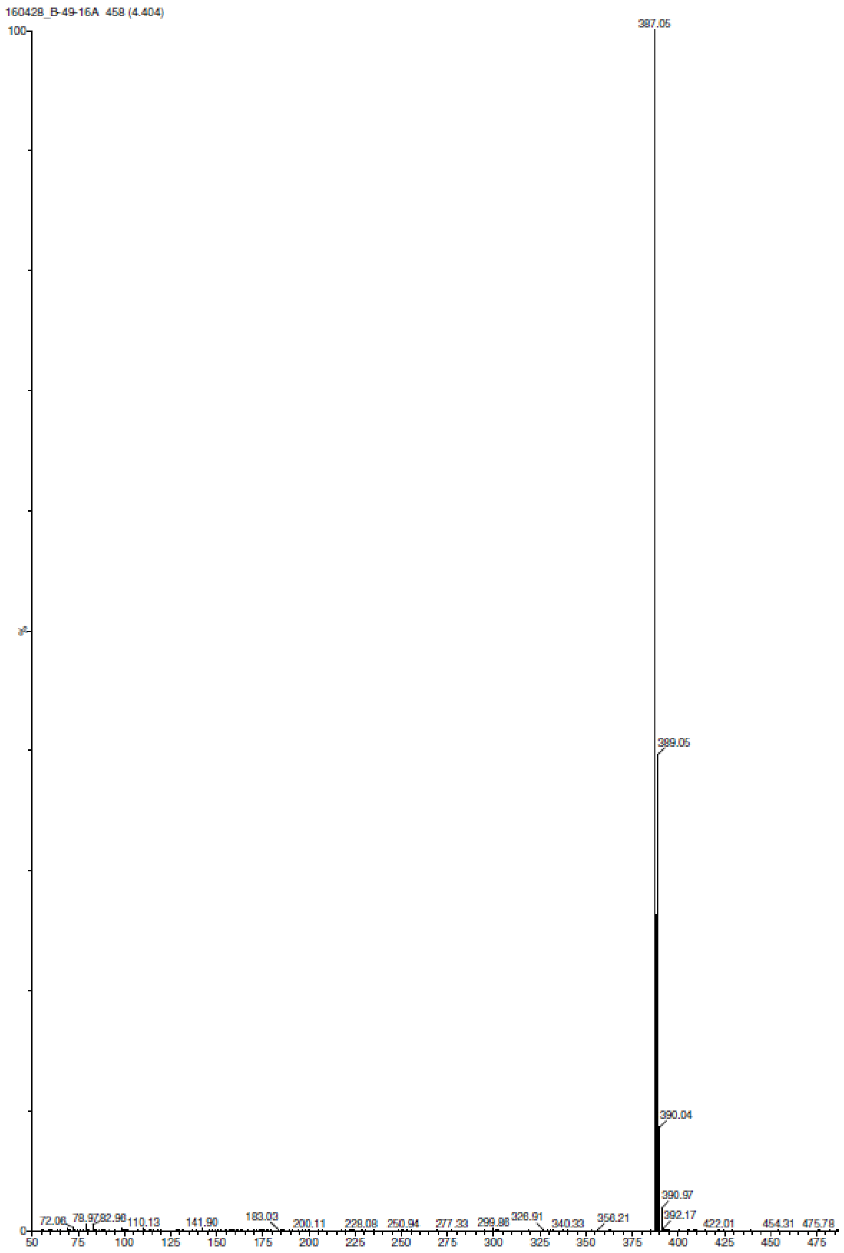
160428_B-49-16A 458 (4.404)
100
%
0
387.05
389.05
390.04
390.97
392.17
72.06
78.97
82.98
110.13
141.90
183.03
200.11
228.08
250.94
277.33
299.86
326.91
340.33
356.21
422.01
454.31
475.78
50
75
100
125
150
175
200
225
250
275
300
325
350
375
400
425
450
475

Figure S8

C2

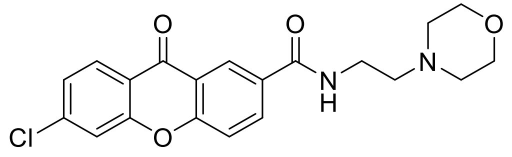
O
O
N
H
N
O
Cl
O

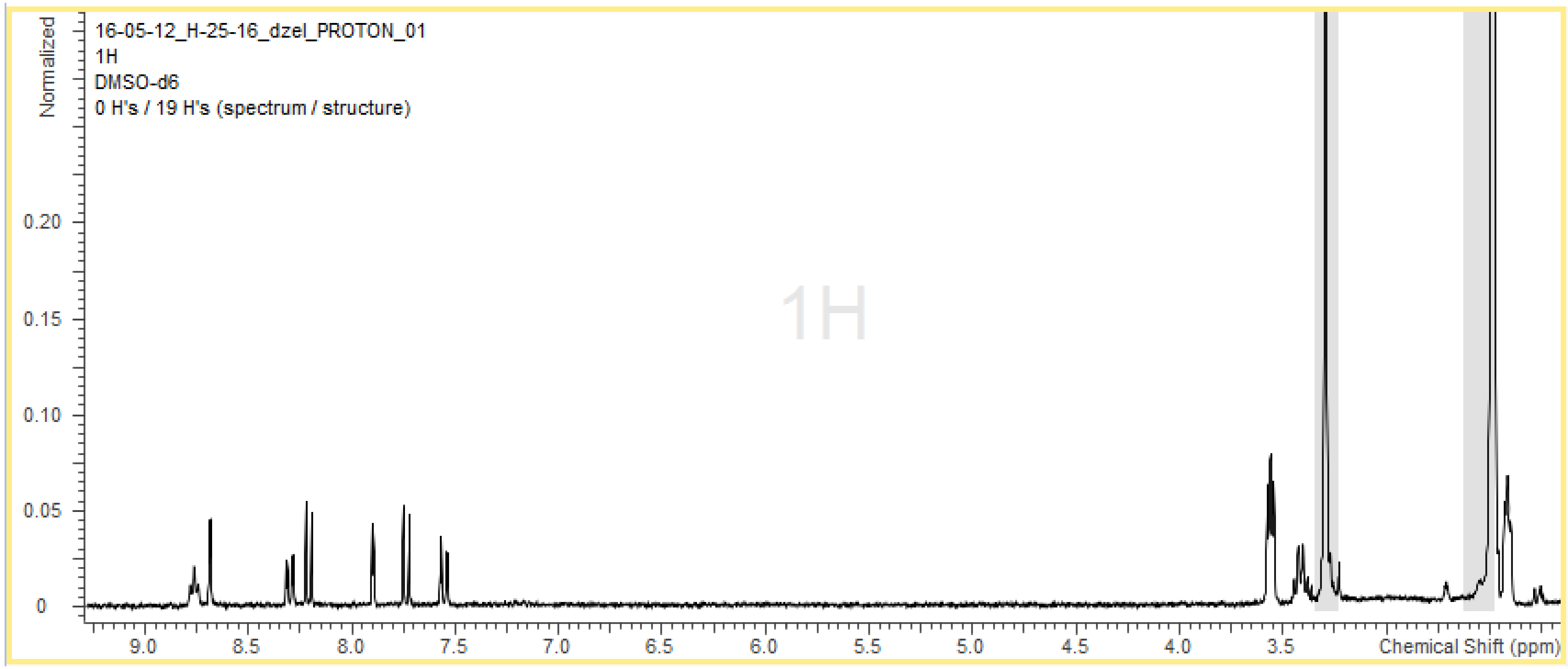
16-05-12_H-25-16_dzel_PROTON_01
1H
DMSO-d6
0 H's / 19 H's (spectrum / structure)
Normalized
0.20
0.15
0.10
0.05
0
1H
9.0
8.5
8.0
7.5
7.0
6.5
6.0
5.5
5.0
4.5
4.0
3.5
Chemical Shift (ppm)

Figure S9

C2

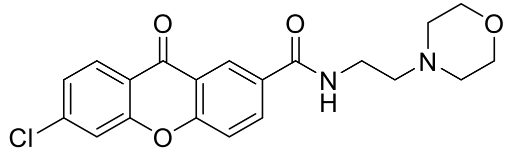

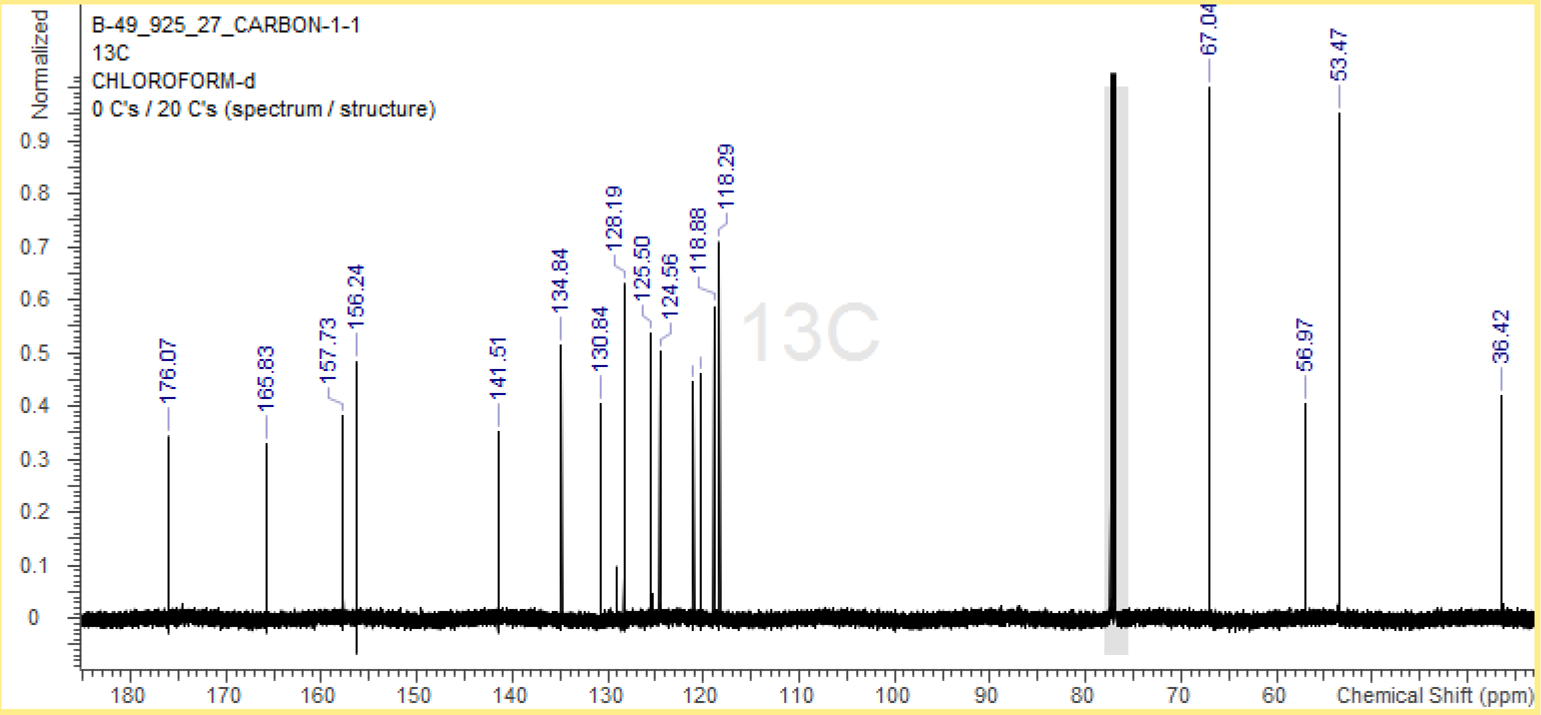

Figure S10

C3

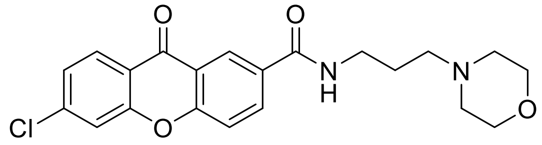

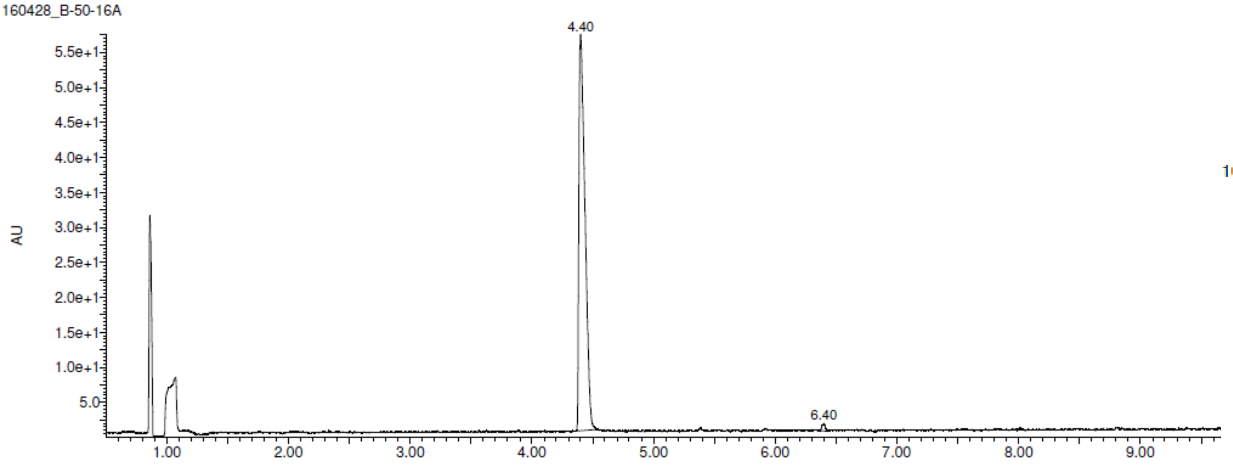

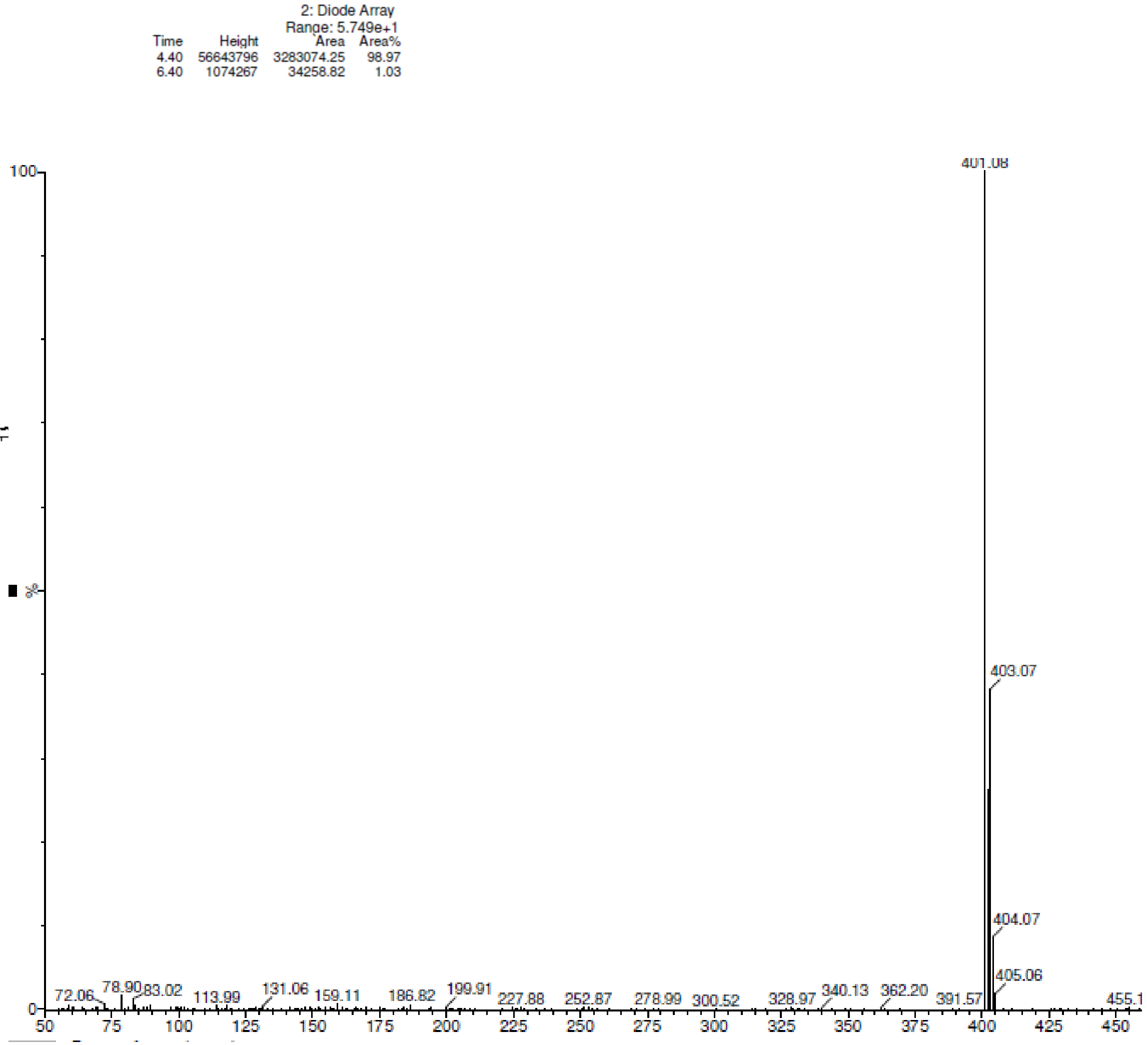

Figure S11

C3

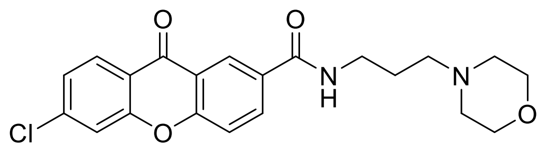

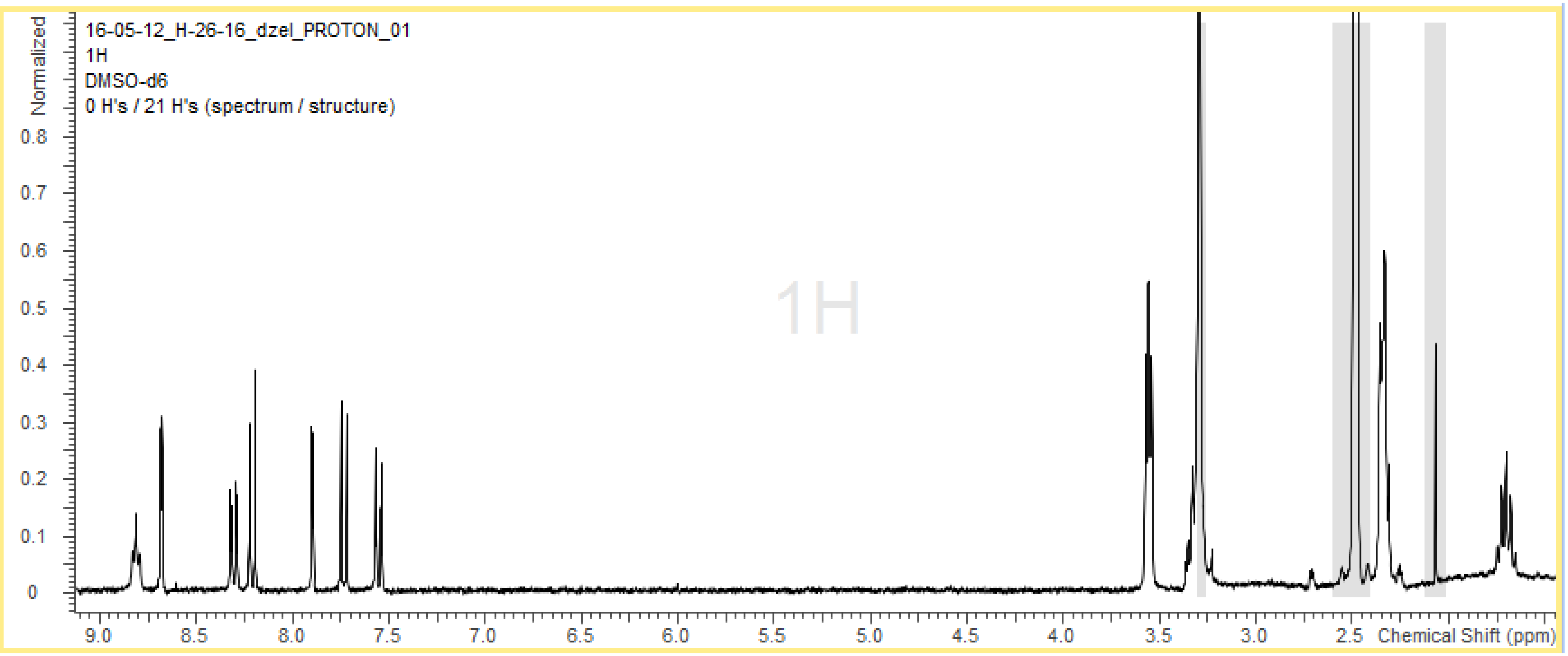

Figure S12

C3

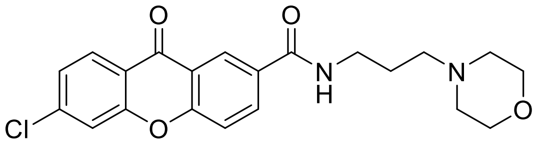

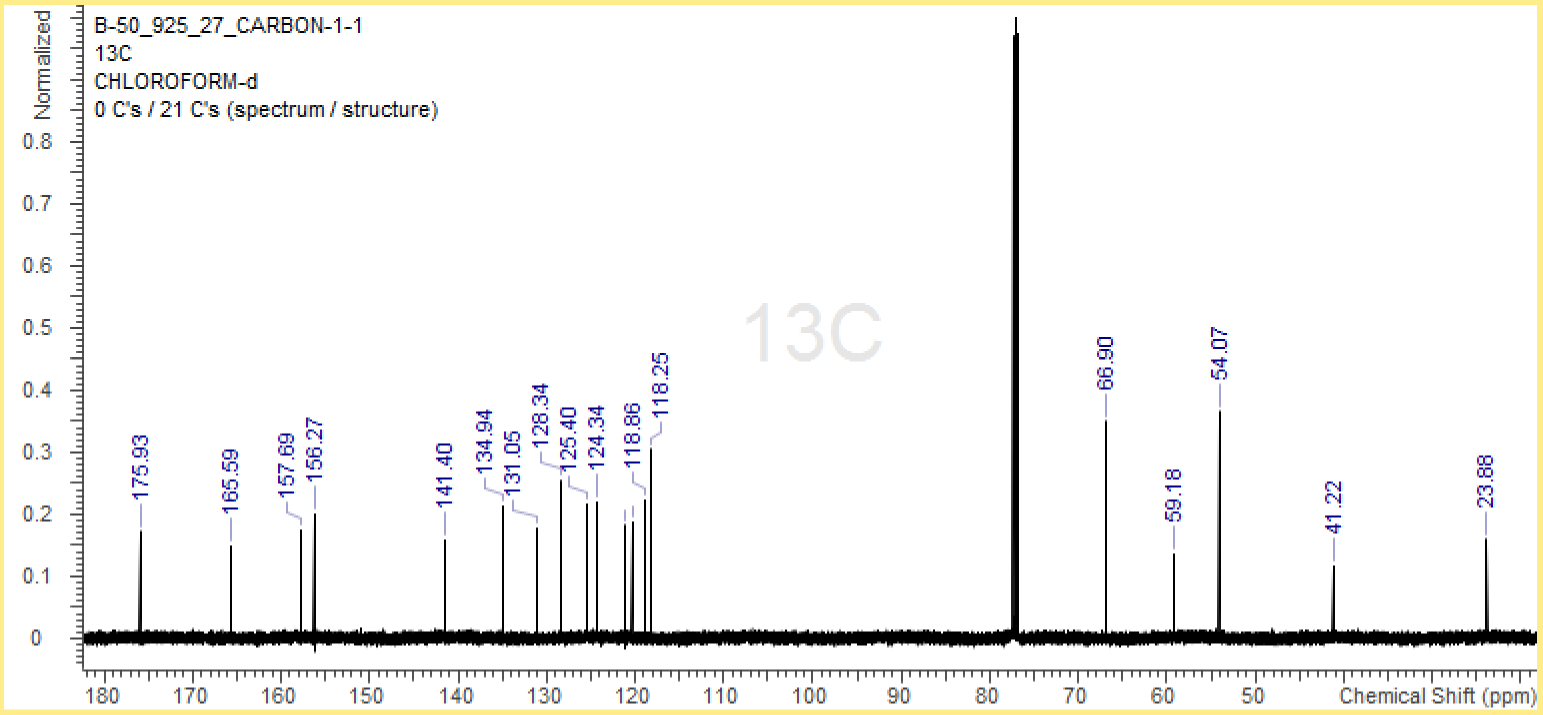

Figure S13

C4

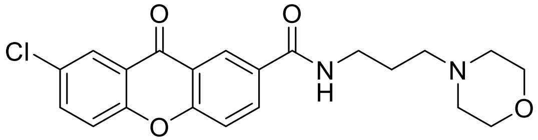

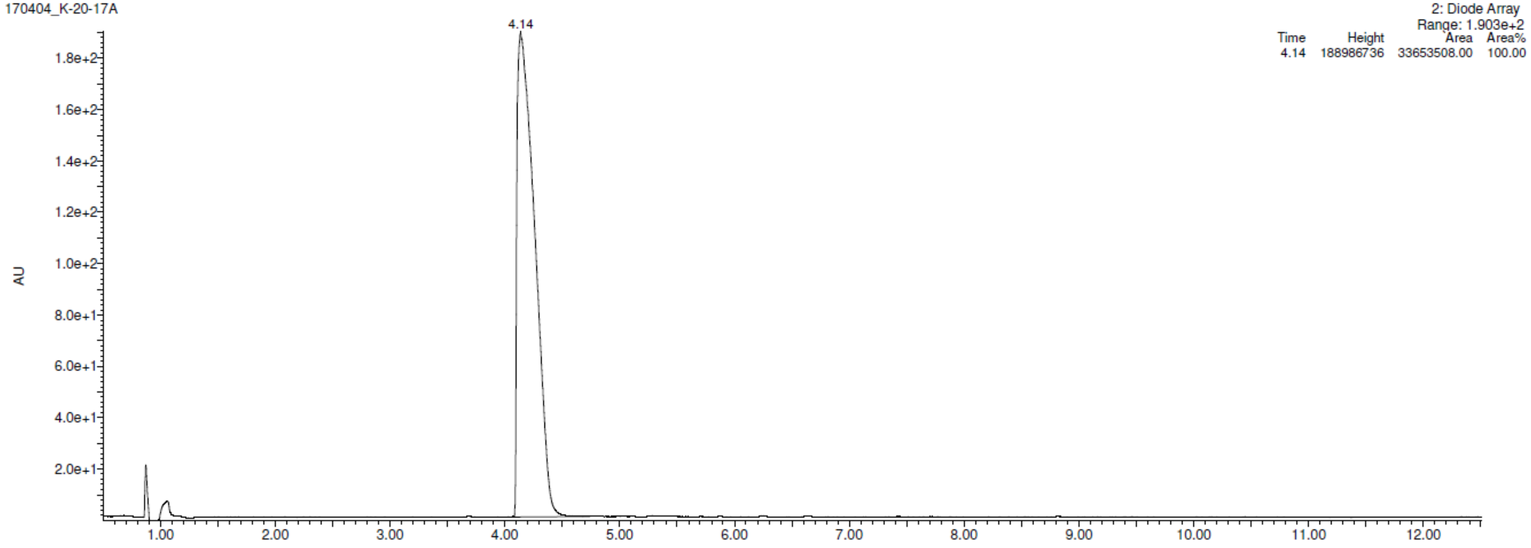

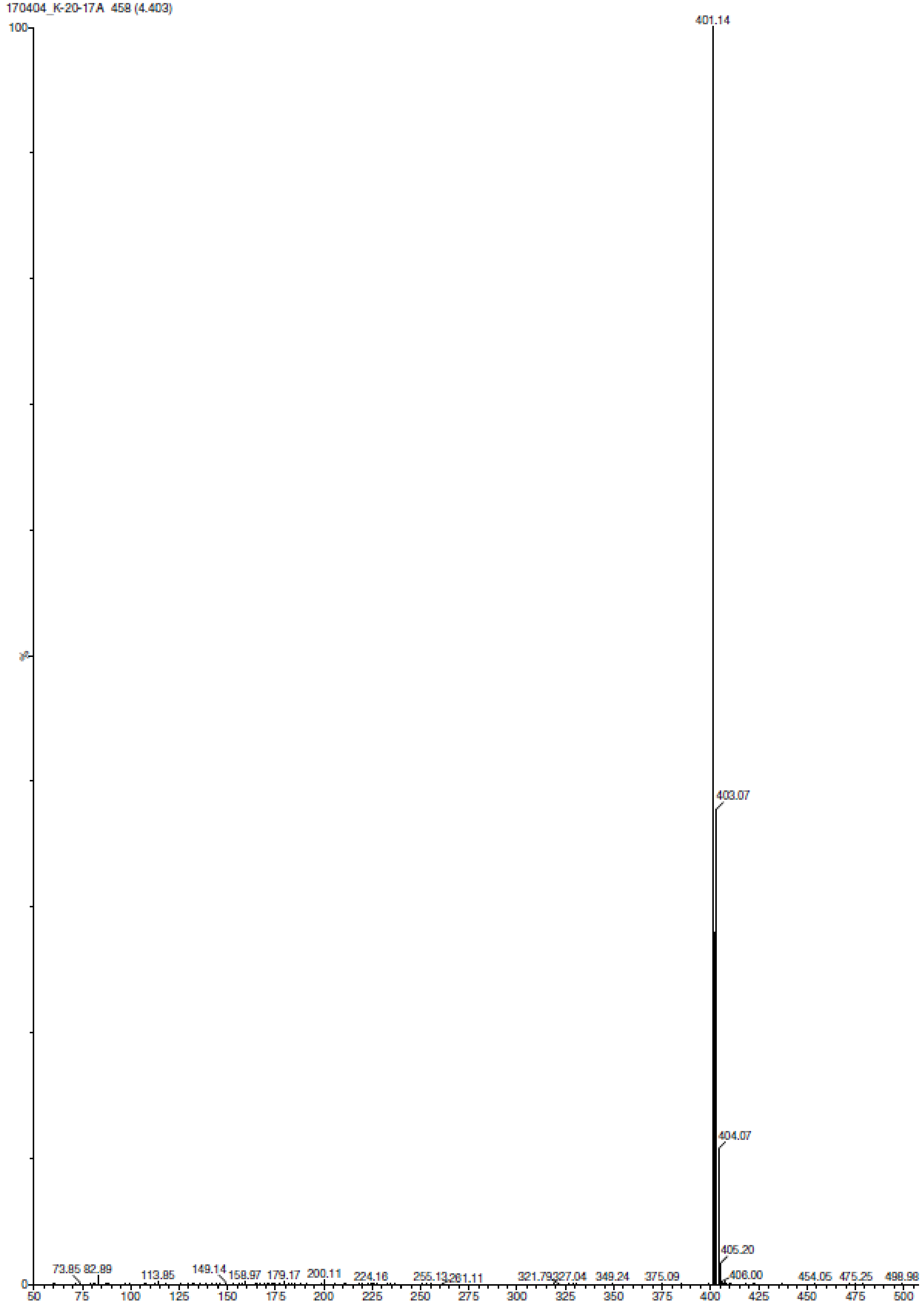

Figure S14

C4

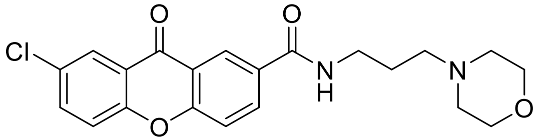

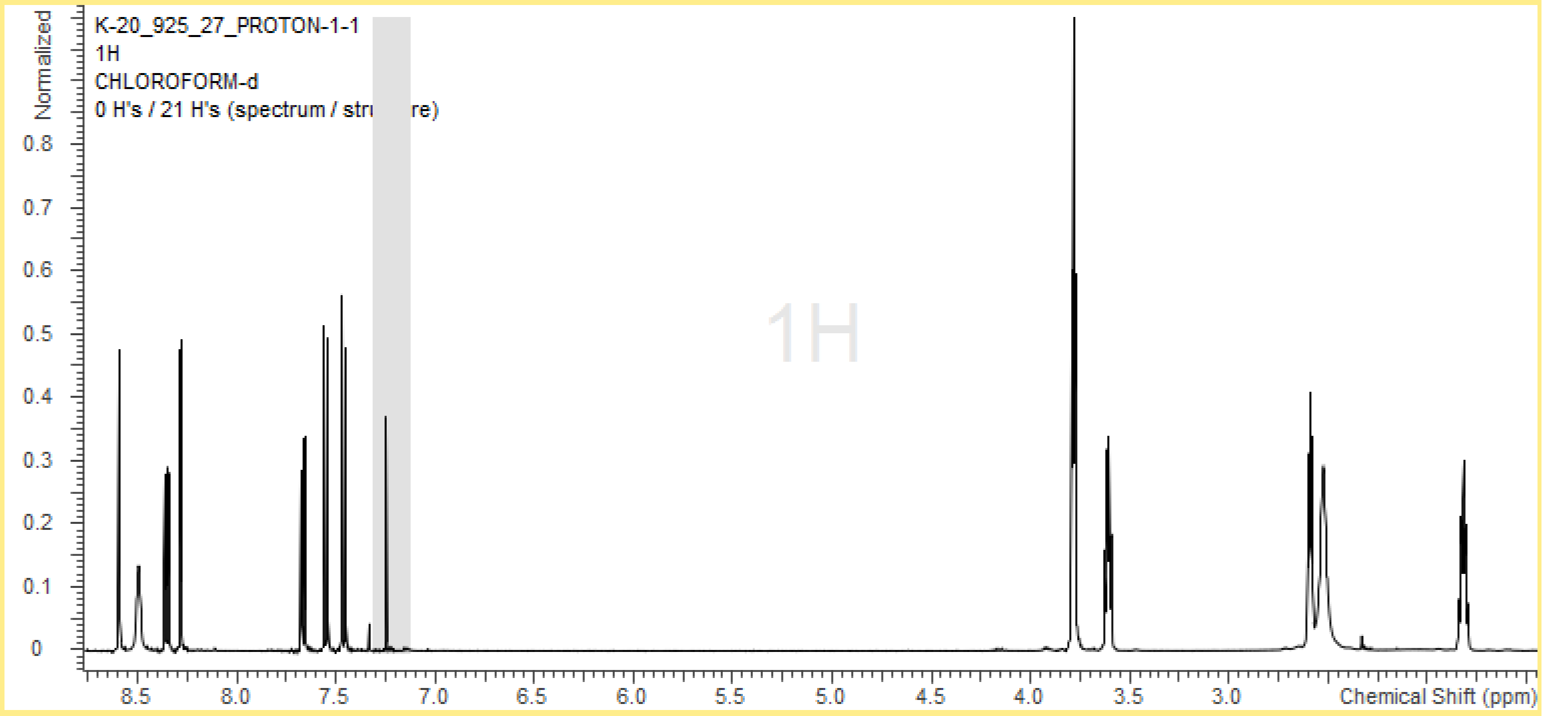

Figure S15

C4

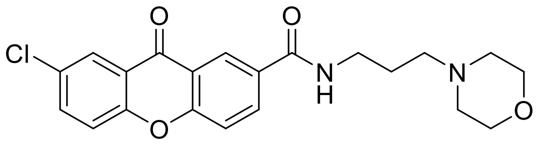

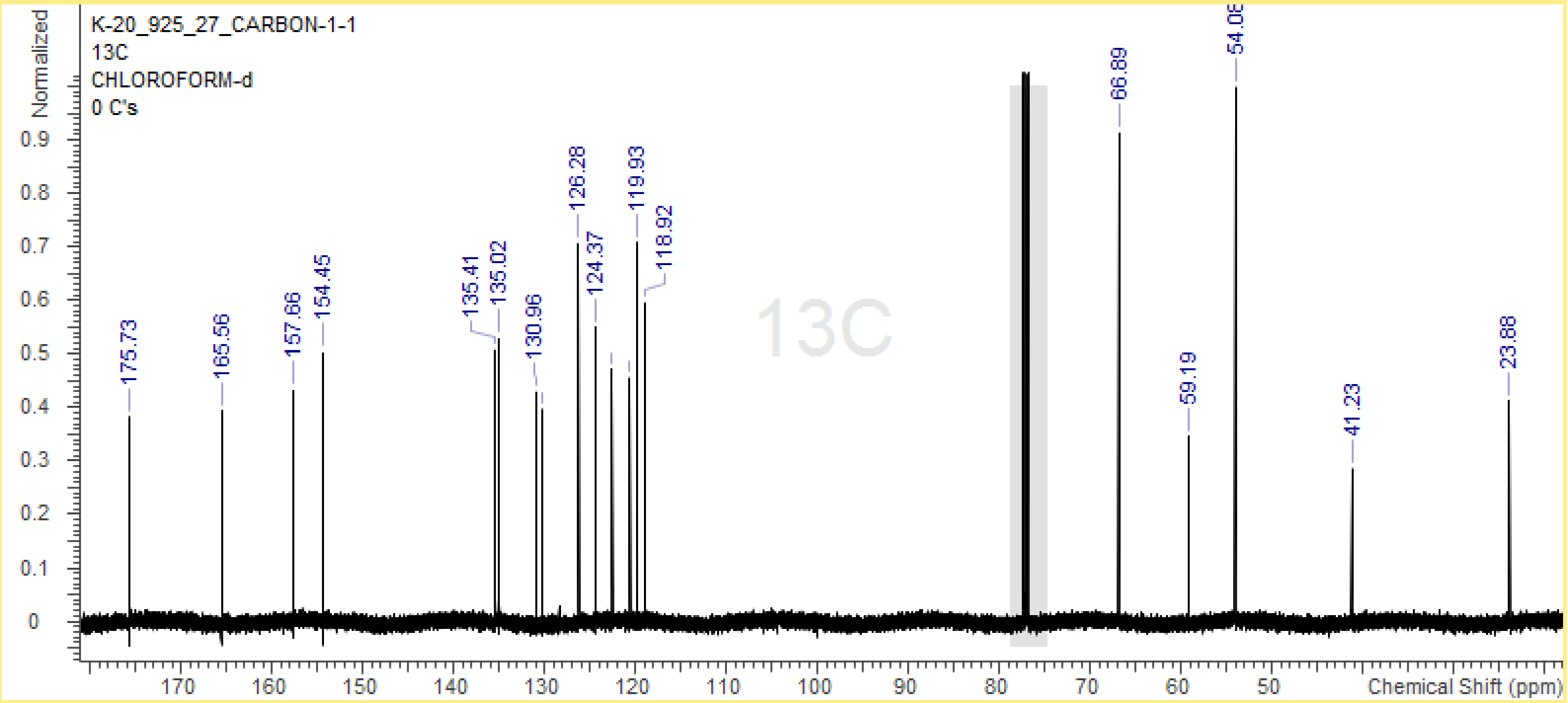

Figure S16

C5

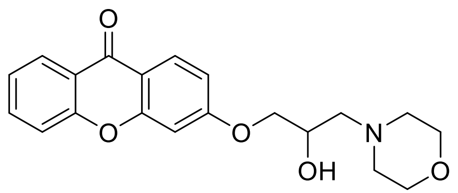

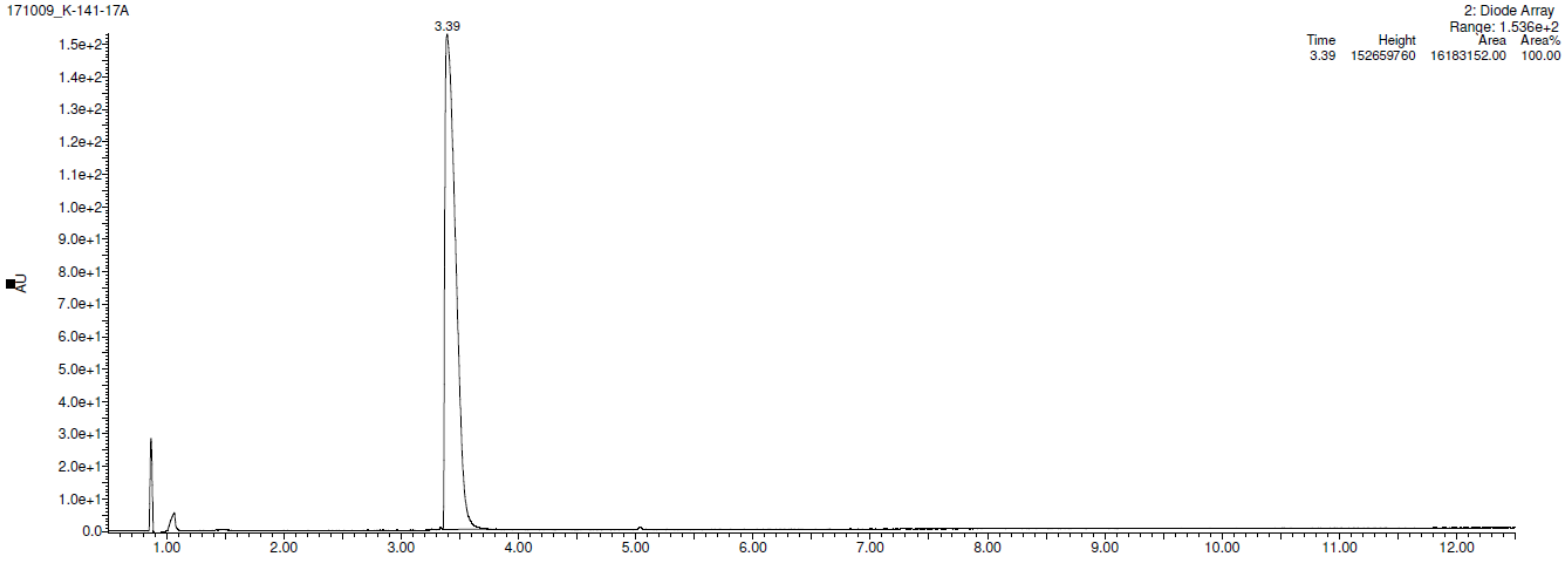

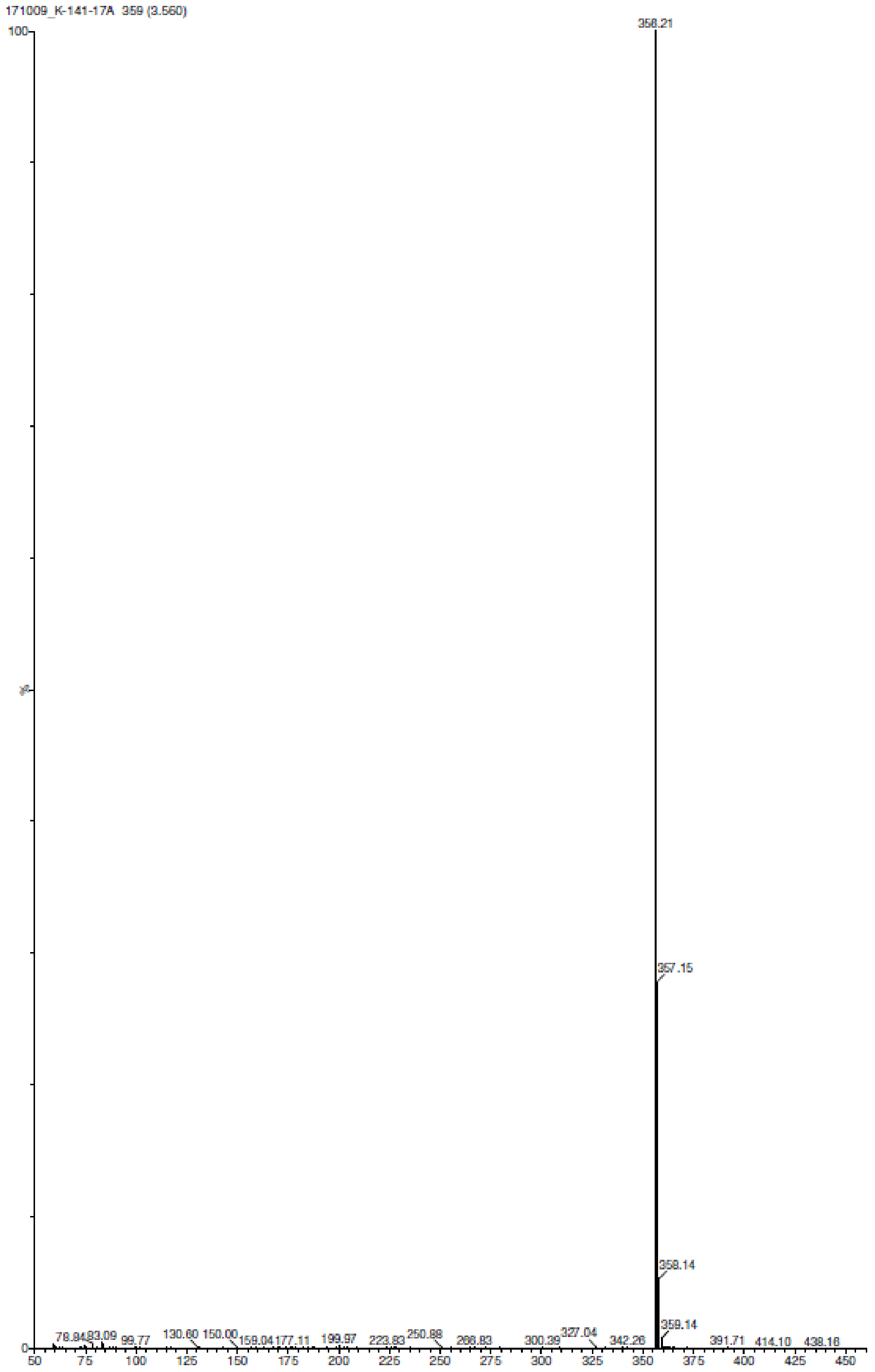

Figure S17

C5

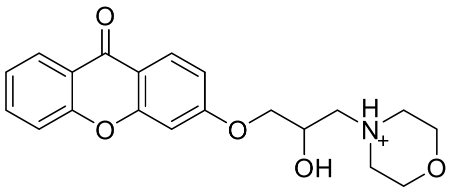

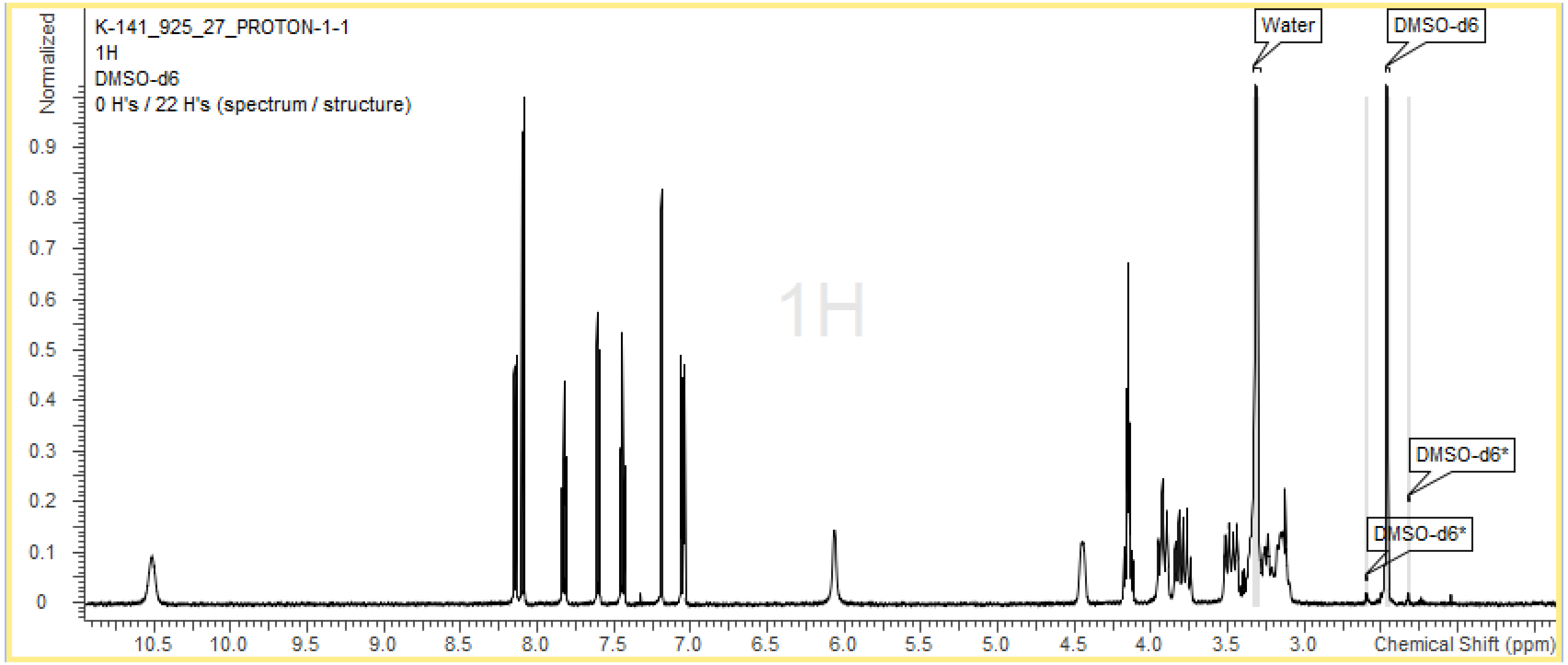

Figure S18

C5

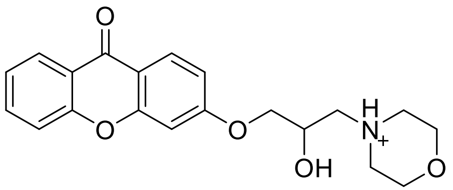

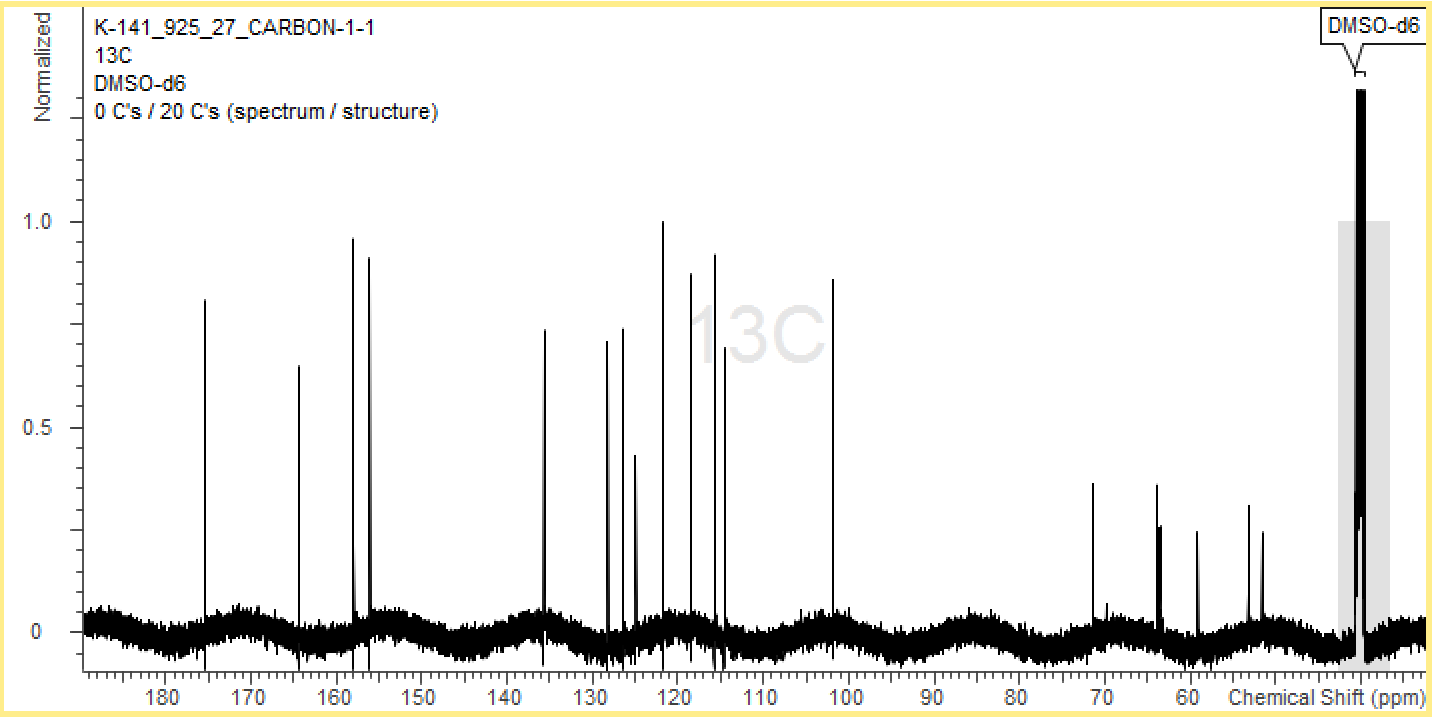

Figure S19

C6

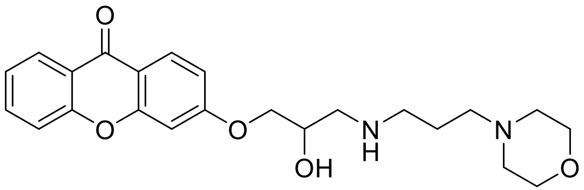

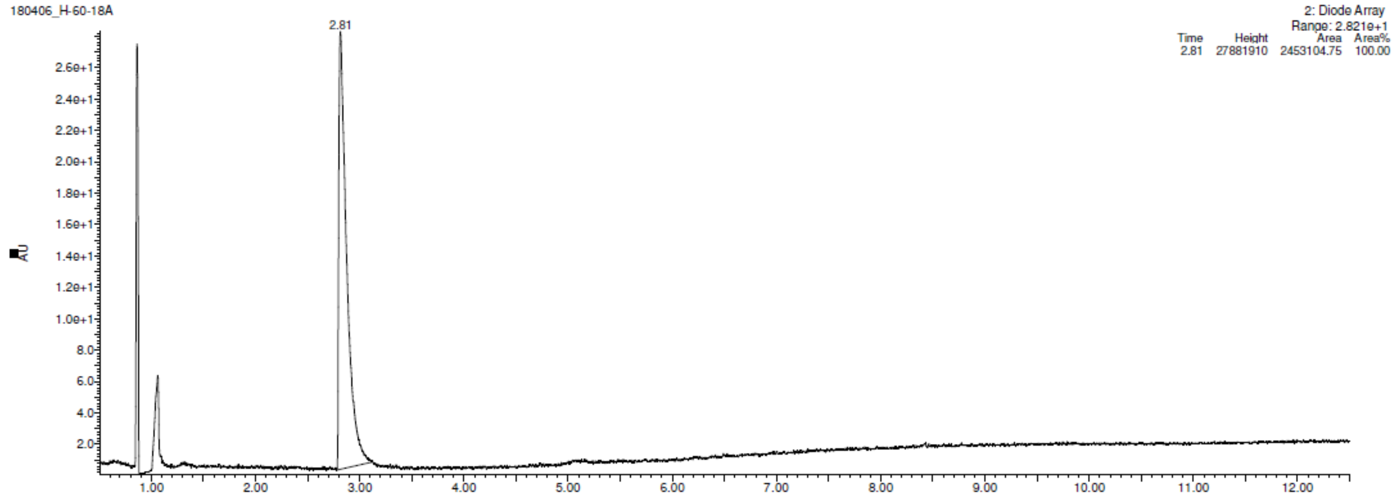

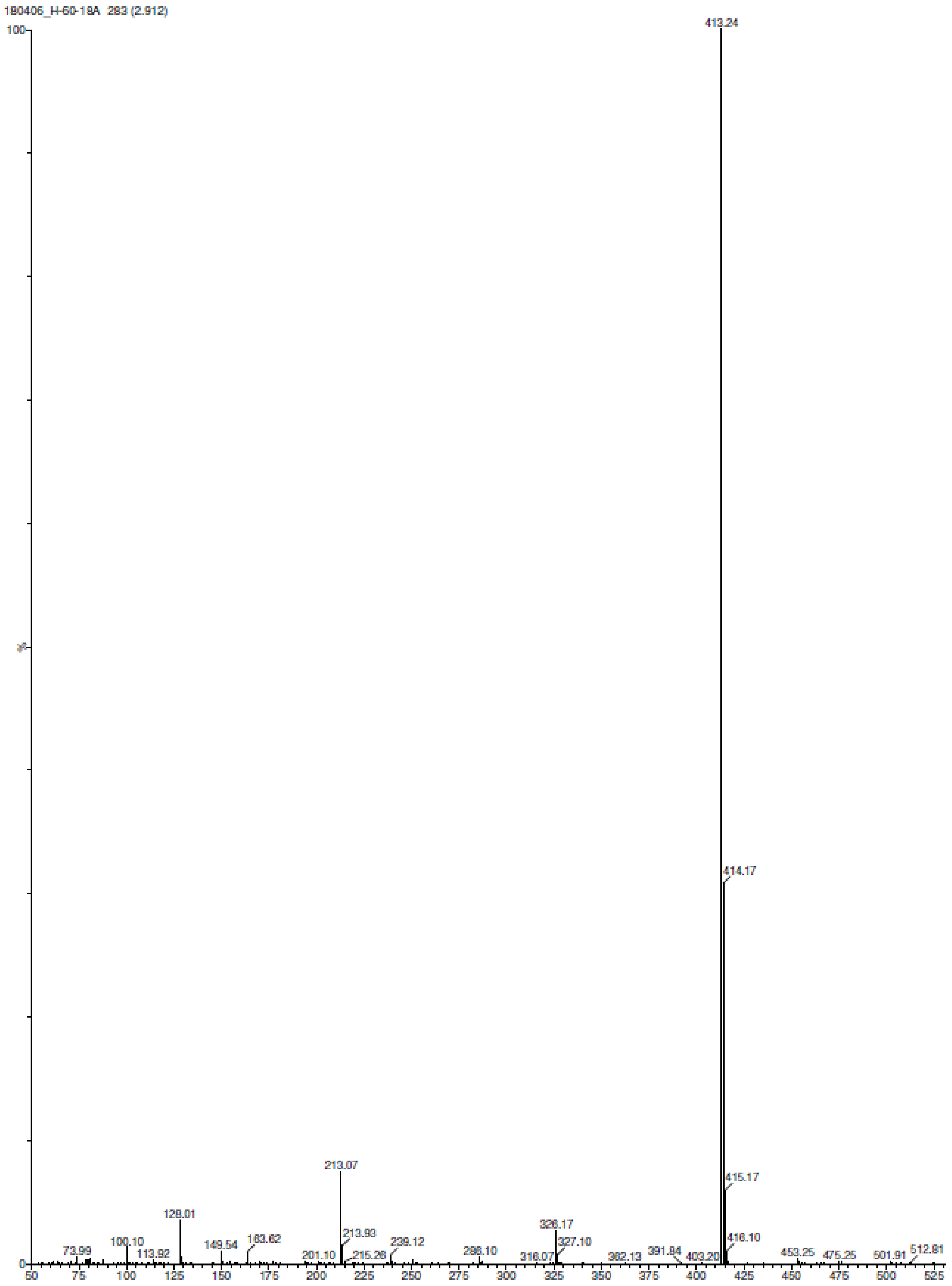

Figure S20

C6

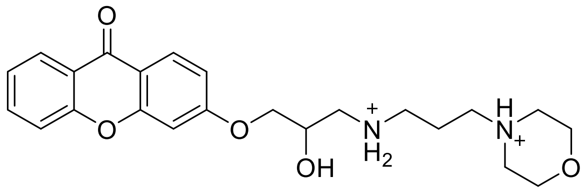

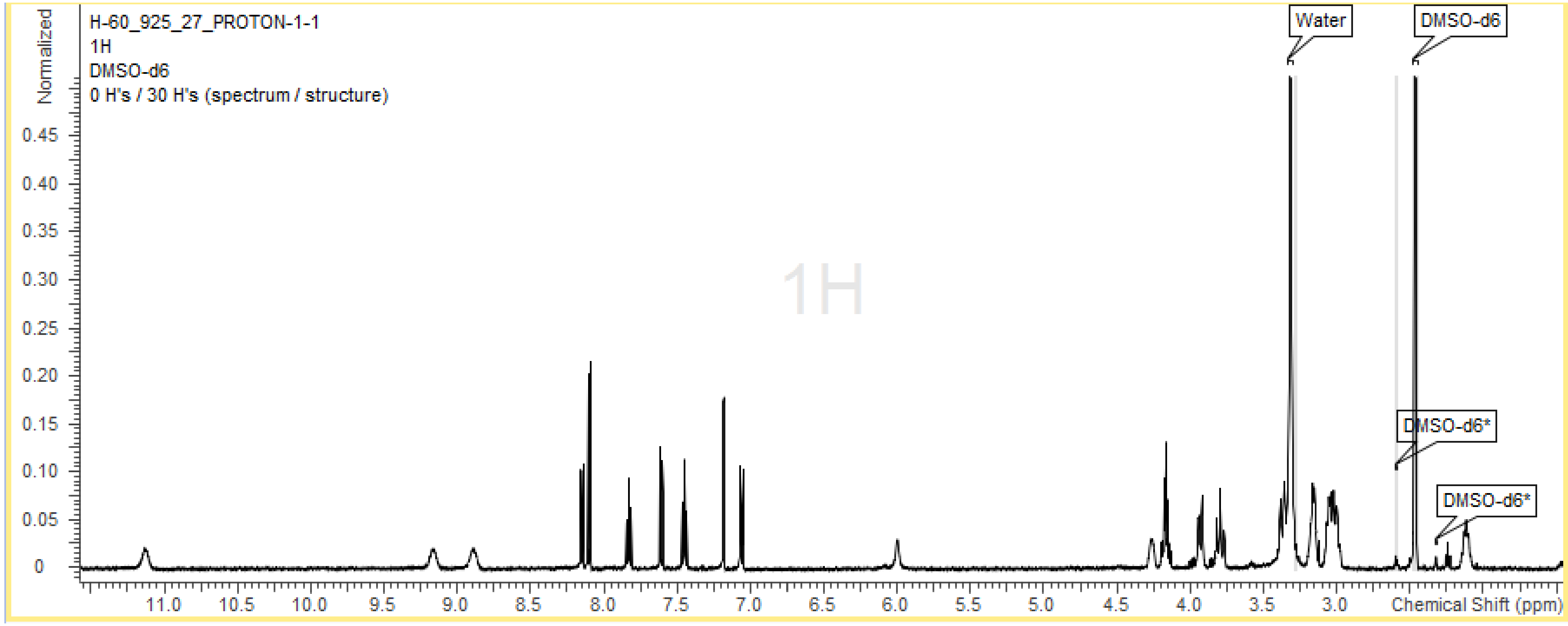

Figure S21

C6

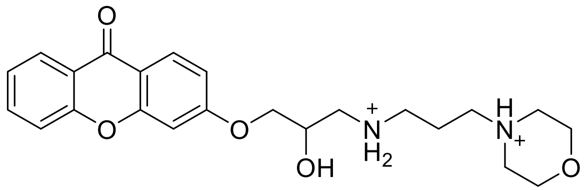

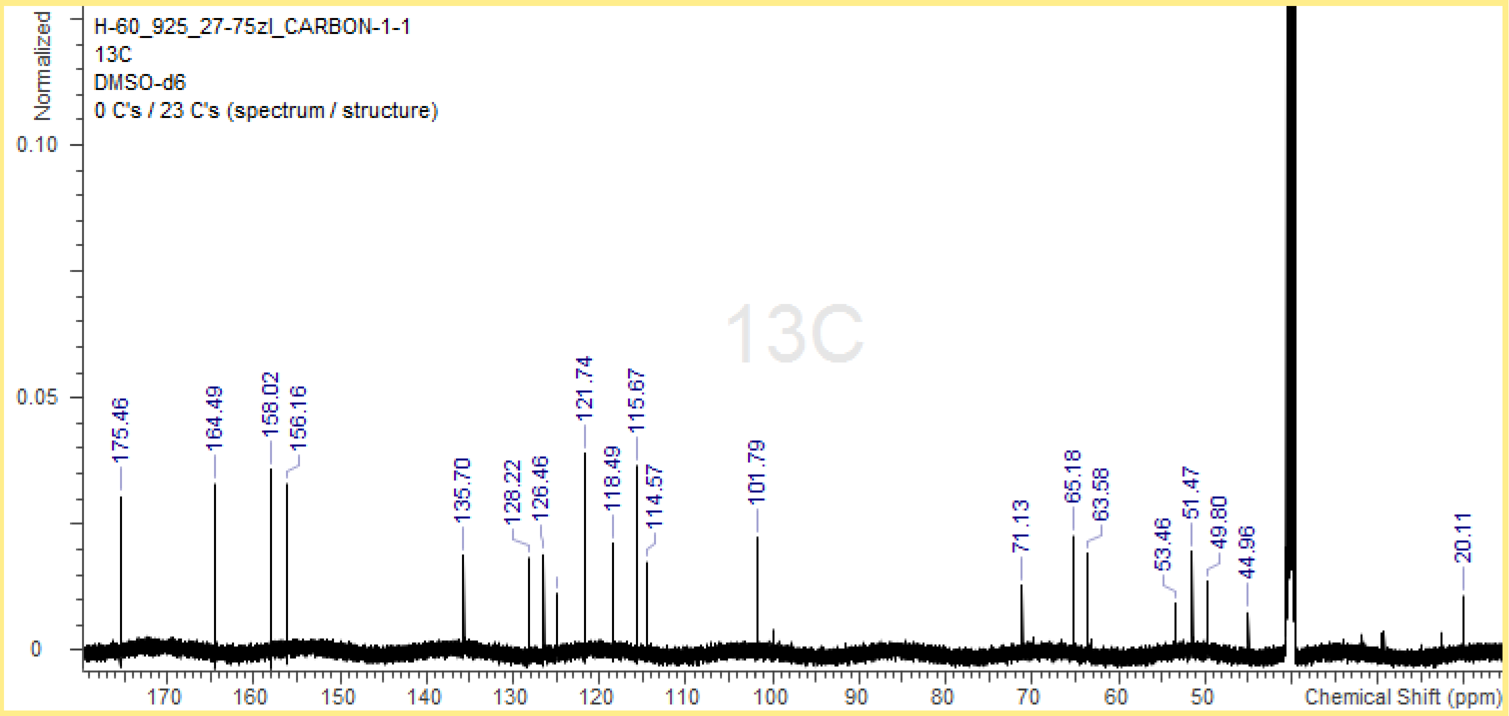

Figure S22

C7

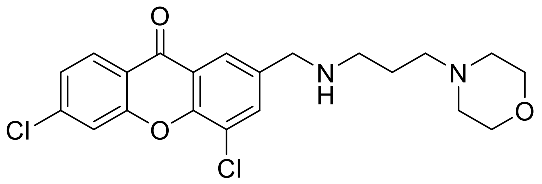

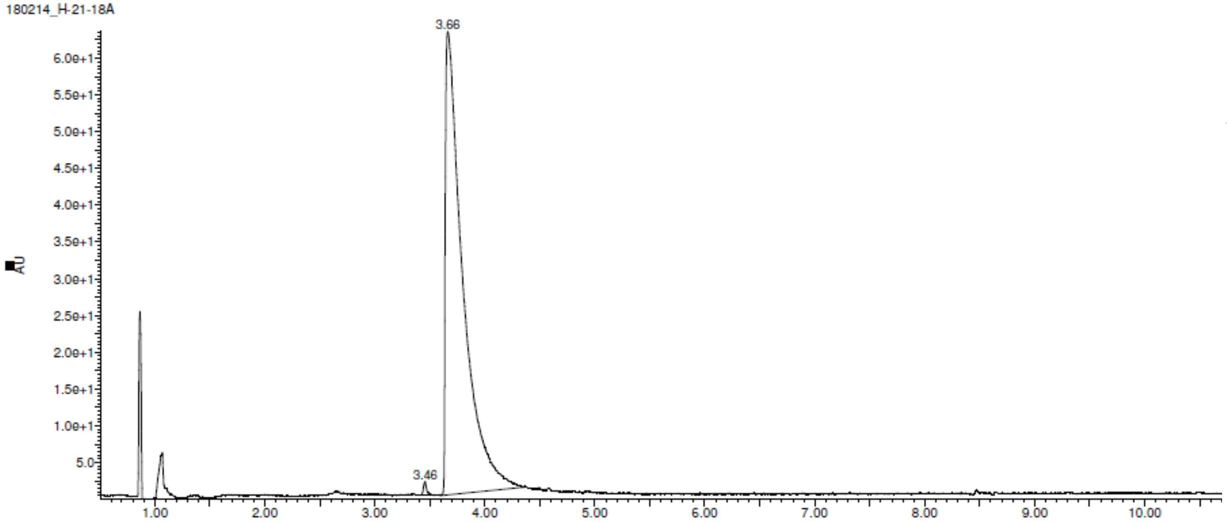

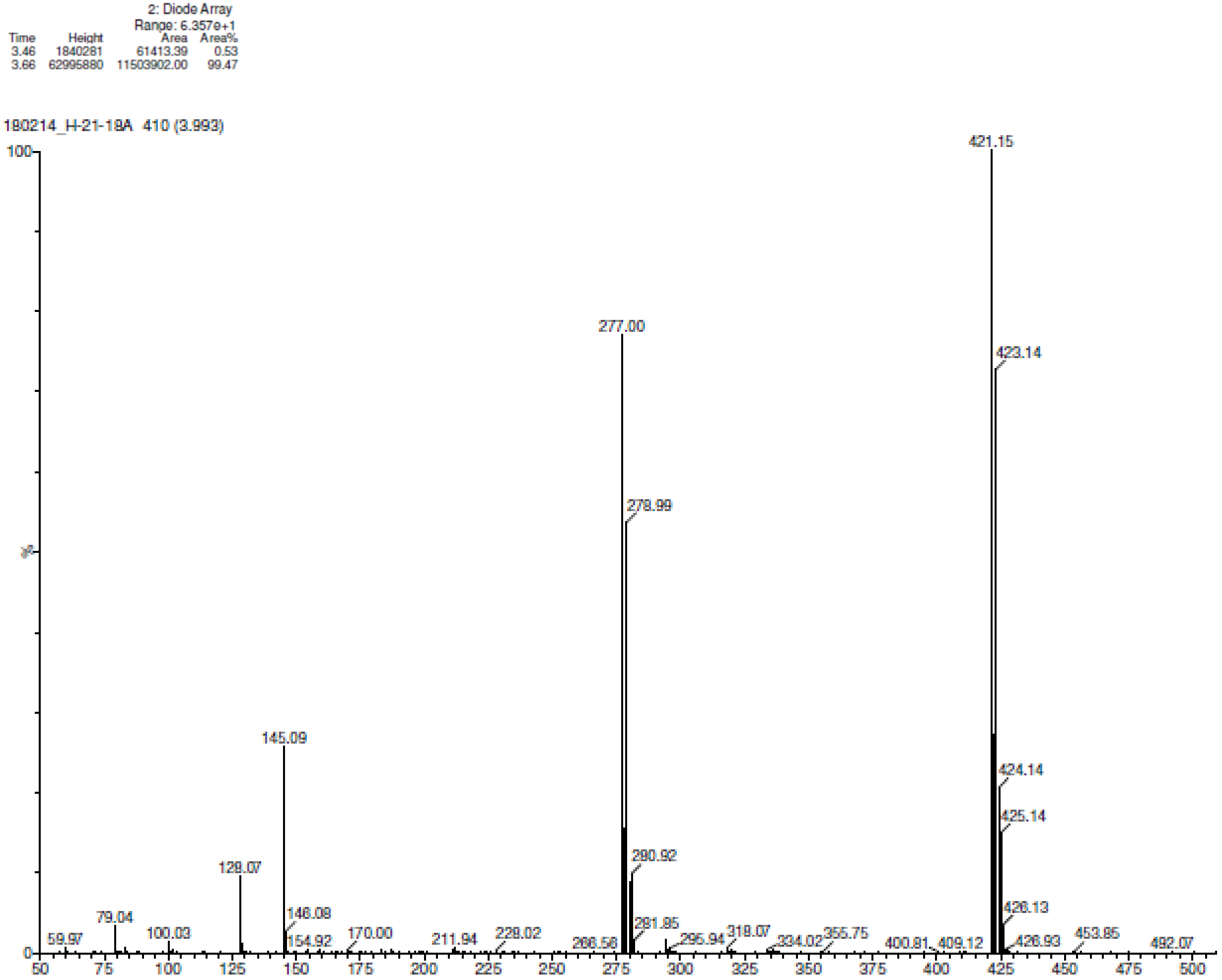

Figure S23

C7

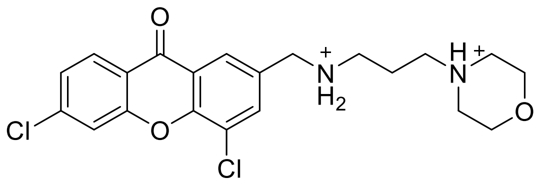

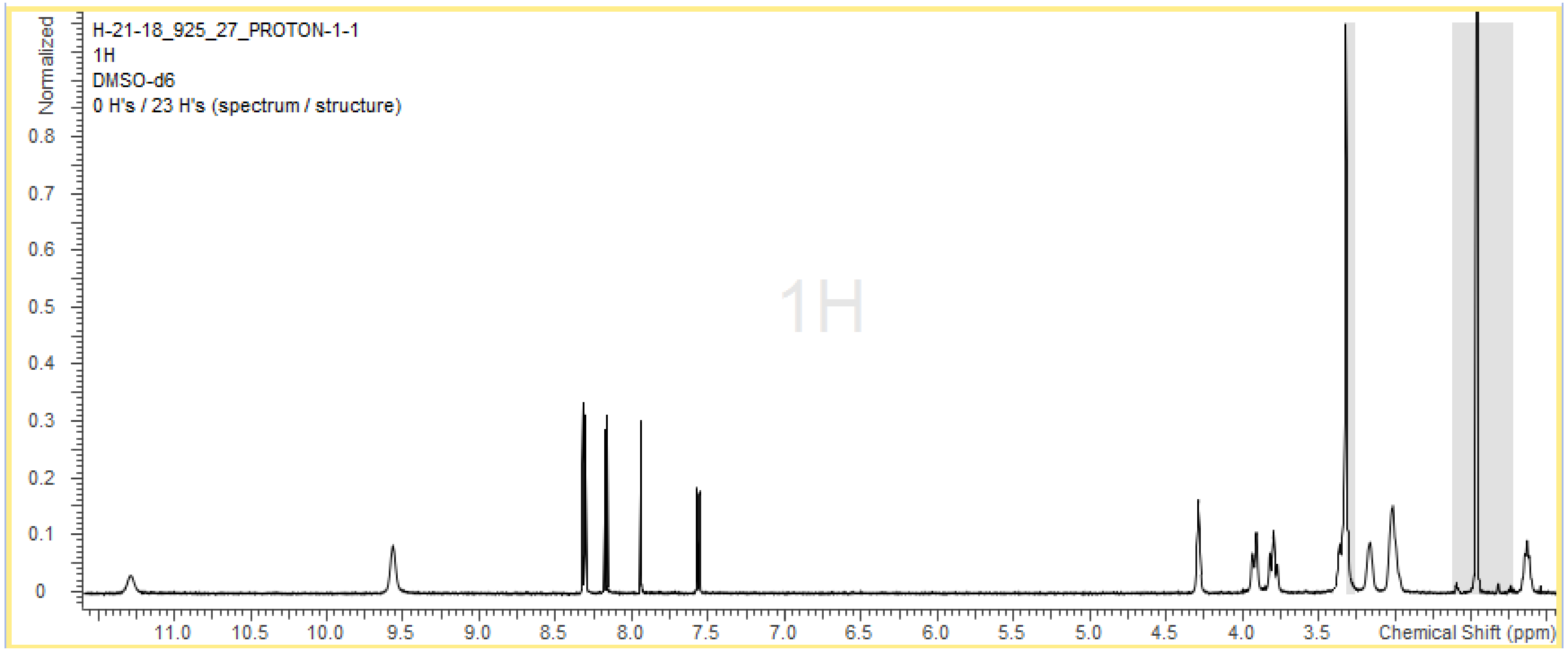

Figure S24

C7

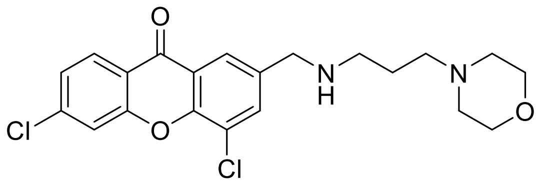

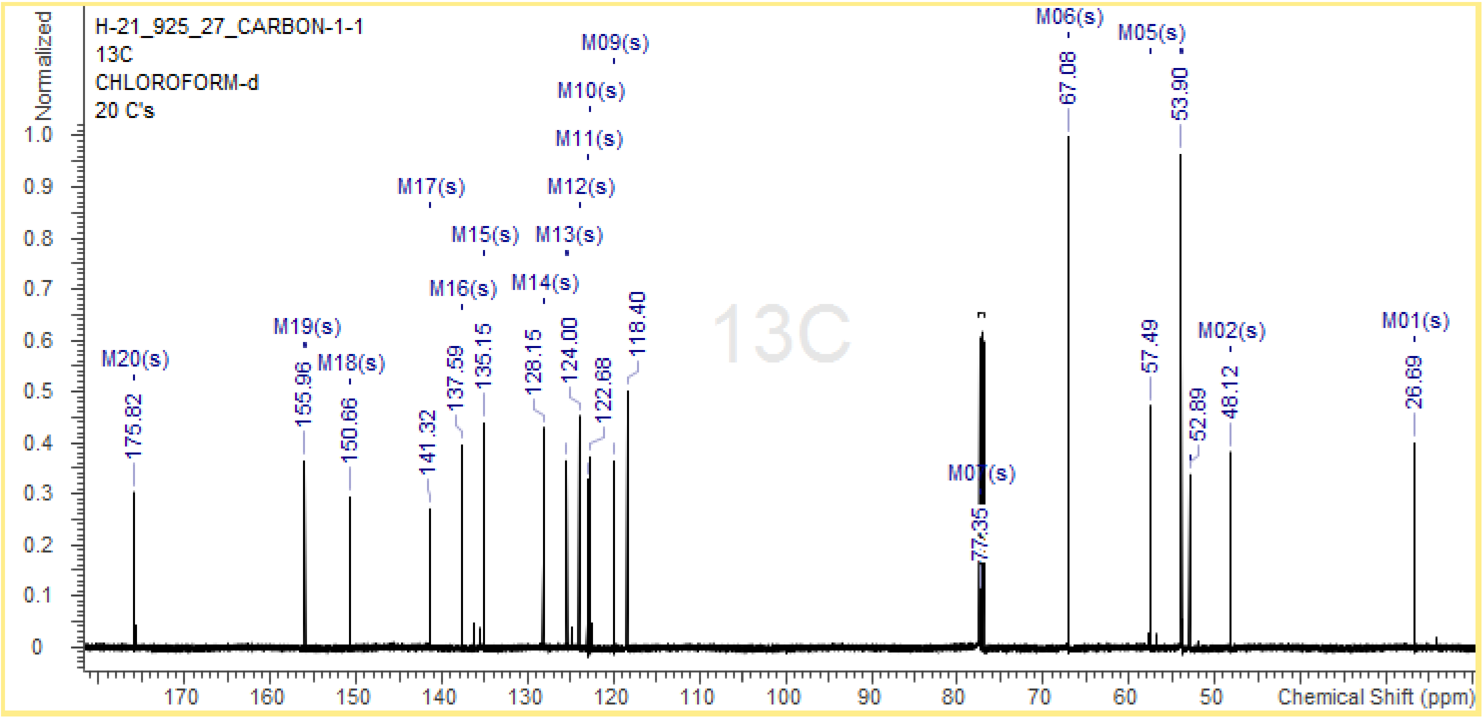

Figure S25

C8

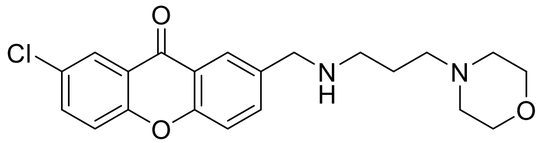

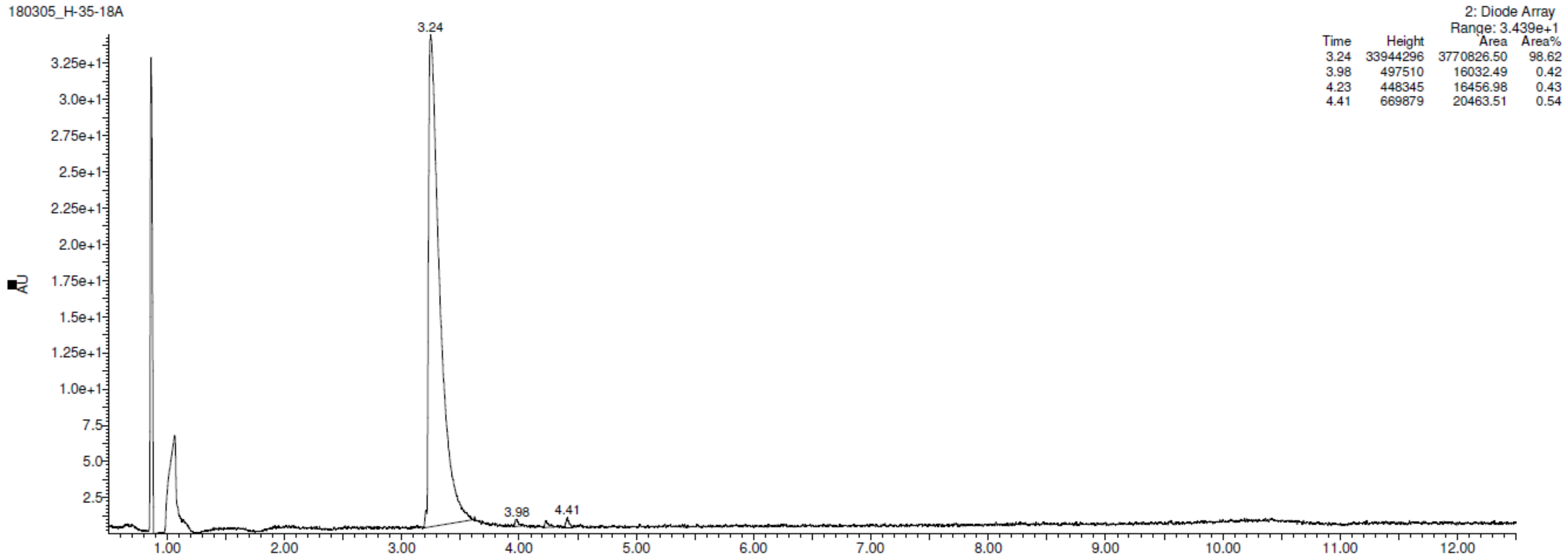

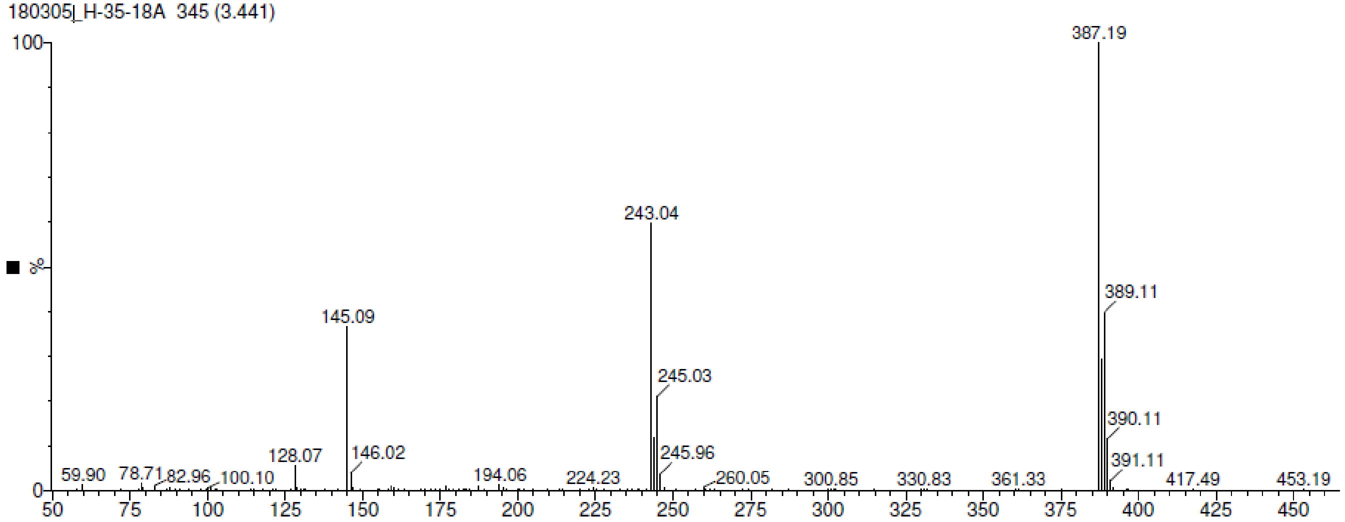

Figure S26

C8

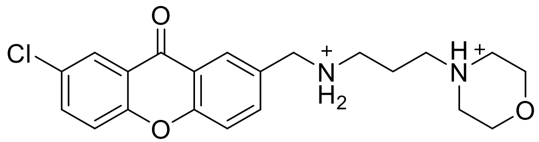

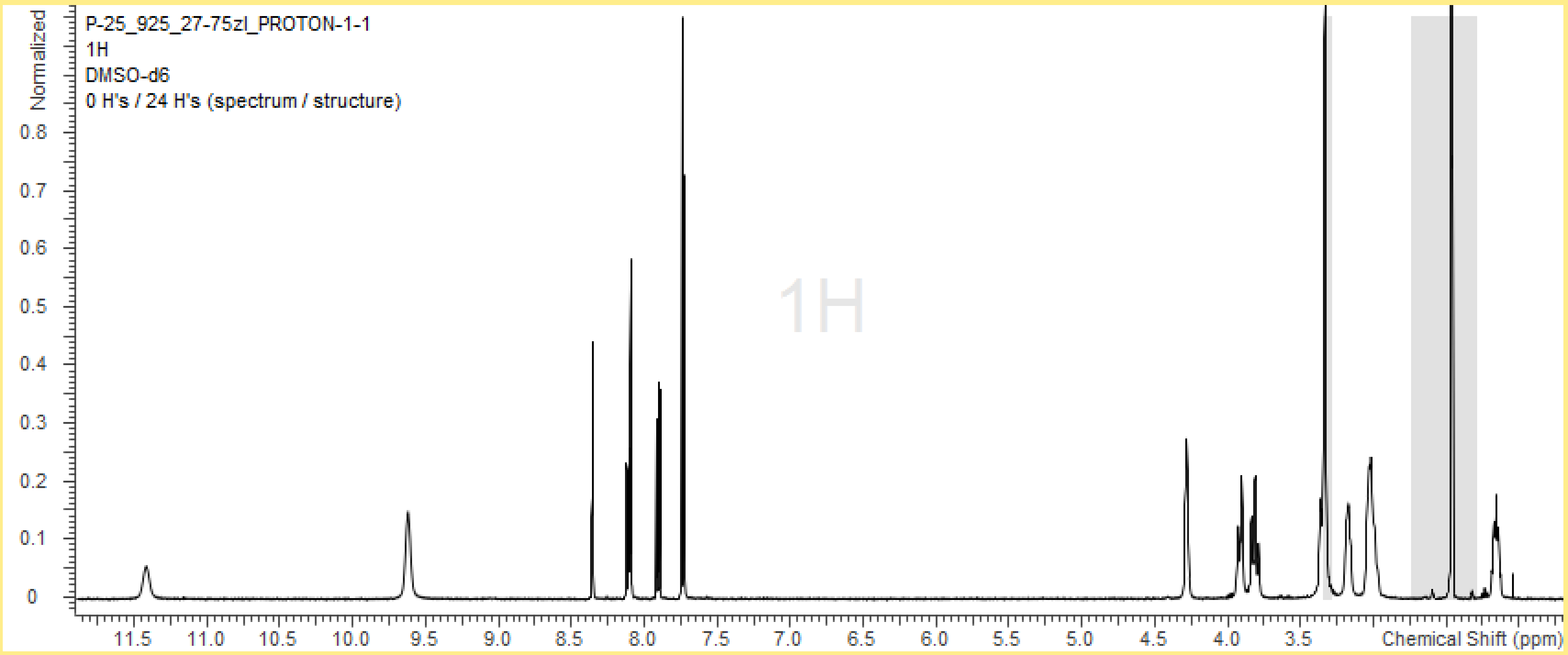

Figure S27

C8

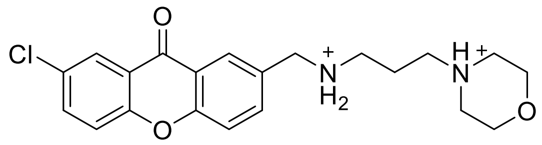

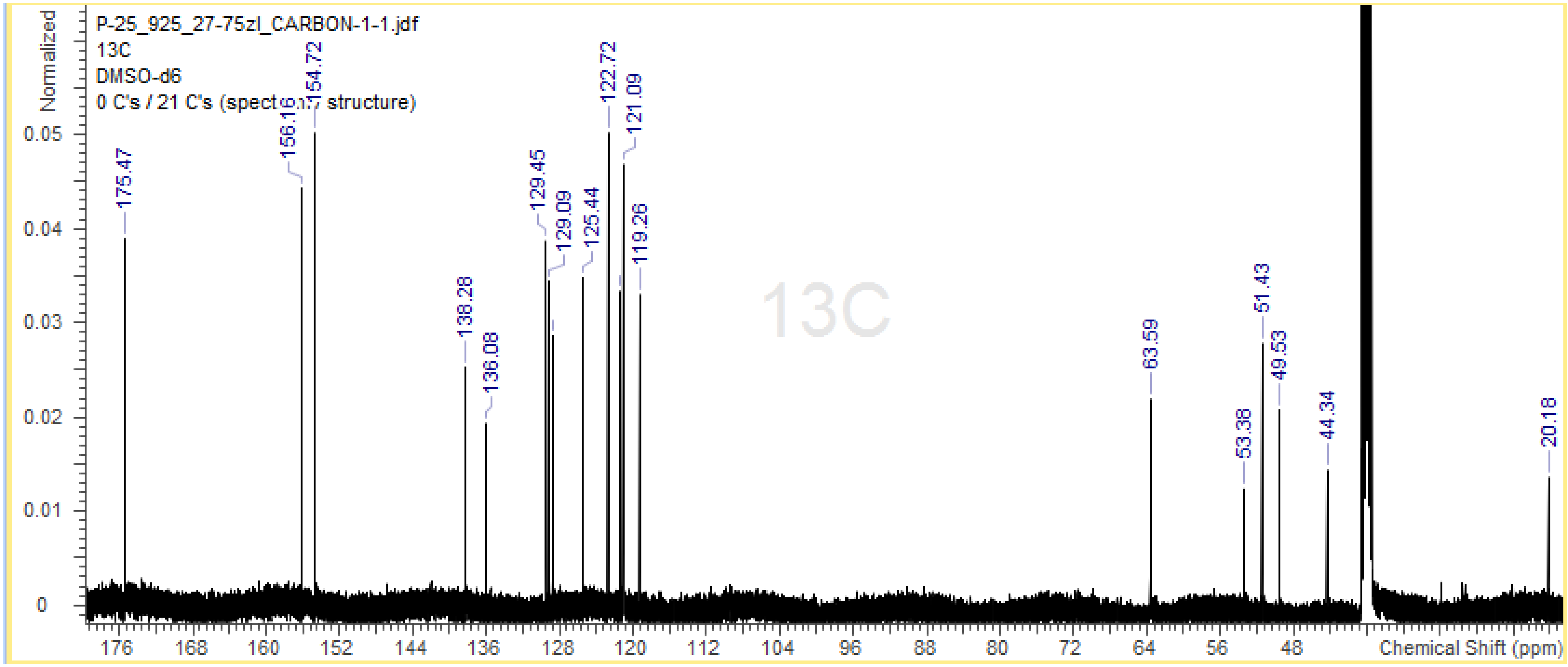

Figure S28

Table S1

L929	IC50 [µM]	IC25 [µM]	GI10 [µM]
CIS	121,71	78,45	52,50
MAG	32,48	15,26	4,92
C8	59,64	32,50	16,21
C7	27,57	16,61	10,04

Table S1. L929 growth inhibition (GI) values in 37°C for 24h
